# Proteome Analysis of Urinary Biomarkers in Acute Hypercoagulable State Rat Model

**DOI:** 10.3389/fmolb.2021.634606

**Published:** 2021-04-30

**Authors:** Jian Jing, Zhenhuan Du, Weiwei Qin

**Affiliations:** Beijing Key Lab of Genetic Engineering and Biotechnology, College of Life Sciences, Beijing Normal University, Beijing, China

**Keywords:** biomarkers, acute hypercoagulable state, blood, urine, proteome

## Abstract

Thrombotic diseases are usually preceded by a hypercoagulable state in the body. This study aimed to screen potential urinary biomarkers for hypercoagulable state based on proteome analysis. Wistar rats were administered with the hemostatic agent etamsylate to establish hypercoagulable state. Urine samples were collected for proteome analysis. We found 20 proteins with levels more than 1.5-fold in difference between control rats and model rats. We searched human homologs of 20 rat proteins and identified 13 human proteins. Of the 13 human homologous proteins, nine were members of human core urinary proteome. Human homologous proteins of differential proteins were highly expressed in 31 human tissues, especially in the kidneys followed by digestive system and reproductive system. Surprisingly, we did not identify known coagulation factors as differential proteins in the urine of model rats. Hypercoagulable state of the body may not involve direct changes in coagulation factors but causes the changes upstream of the coagulation cascade system. Common differential urinary proteins between different hypercoagulable states suggest some common pathways in the formation of hypercoagulable states and may serve as potential biomarkers for the prevention and treatment of thrombotic diseases.

## Introduction

Biomarkers are widely used to indicate the changes associated with pathological processes (Jing and Gao, 2016; Jing and Gao, 2018). Urine proteome has emerged as a novel approach to biomarker discovery (Gao, 2013; Li et al., 2014). Notably, a variety of factors such as gender, age, exercise, and diet affect urine proteome (Wu and Gao, 2015). Furthermore, urine could be collected in large quantities in a noninvasive and continuous manner (Jing and Gao, 2018).

Thrombus involves a series of pathological processes. The early stage of thrombus is known as “hypercoagulable state,” which is defined as a group of inherited or acquired conditions associated with predisposition to thrombosis, and the pathogenesis mechanism remains unclear (Heit, 2007; Mitchell et al., 2007). The establishment of the hypercoagulable state model will be helpful to understand the underlying mechanism and develop effective prevention and intervention approaches for thrombotic diseases. Etamsylate (ETSL) is a hemostatic drug (Schulte et al., 2005). ETSL inhibits the biosynthesis and action of prostaglandins to regulate platelet aggregation, vasodilation, and capillary permeability (Kovács and Falkay, 1981).

This study aimed to screen potential urinary biomarkers for hypercoagulable state based on proteome analysis. Wistar rats were administered with ETSL to induce acute hypercoagulable state. Urine samples were then collected for proteome analysis. The differential proteins were then analyzed by bioinformatics analysis.

## Materials and Methods

### Animals

**FIGURE 1:**
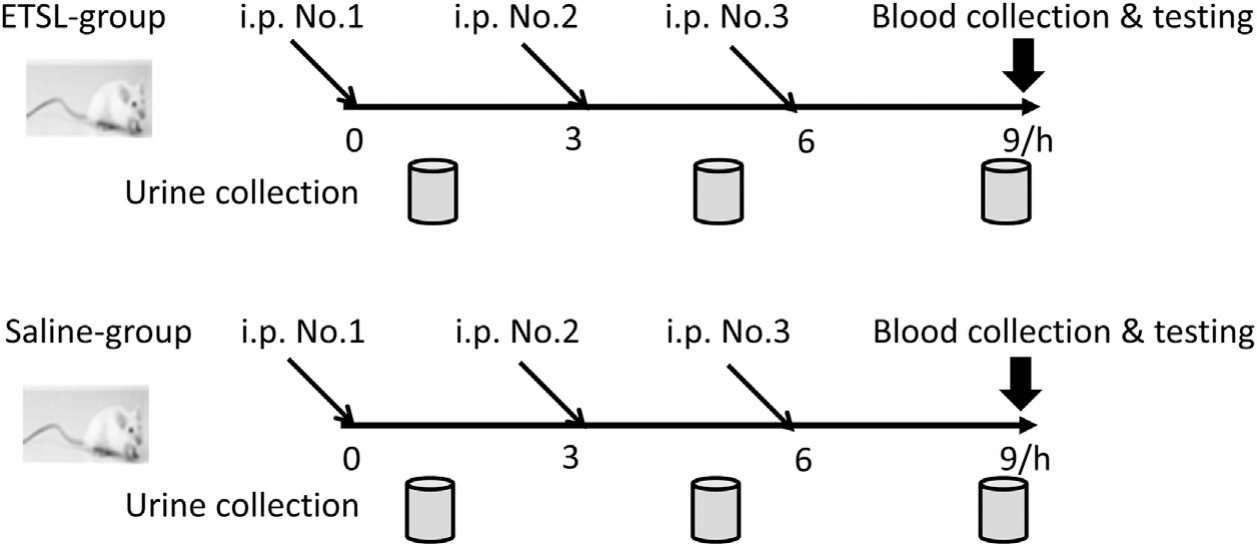
Schematic representation of ETSL-induced rat model of hypercoagulable state. i. p., intraperitoneal injection; ETSL group, group with i. p. of ETSL drug; Saline group, control group with i. p. of normal saline; i. p. No. 1, i. p. for the first time; i. p. No. 2, i. p. for the second time; i. p. No. 3, i. p. for the third time.

All animal protocols were approved by Animal Ethics Committee, College of Life Sciences, Beijing Normal University (Approved ID: A02-2015-008), and all efforts were made to minimize the suffering of the animals. Specific pathogen-free male Wistar rats (weight 200 g) were purchased from Vital River Laboratory (Beijing, China) and maintained in standard conditions. Rats were acclimated for a week and then divided into two groups randomly (*n* = 10). Rats in the model group received intraperitoneal injection of ETSL (from Jinyao Pharmaceutical, Tianjin, China) at the dose of 250 mg/kg body weight three times at 0, 3, and 6 h, and rats in control group received equal volume of saline. Urine samples were collected after the first injection in the 1.5- to 9-h period and preserved at −80°C for proteome analysis (Figure 1). At 3 h after the third injection, blood samples were taken from the medial canthus vein to measure activated partial thromboplastin time (APTT) and coagulation time (CT).

### APTT and CT Measurements

APTT was measured as described previously (Shi and Gilbert, 2003). Briefly, 100 μL plasma was used and APTT was determined using commercial kit (Taiyang Biotech., Shanghai, China). CT was measured by test tube method. Briefly, 25 μL blood sample was placed in a test tube and the tube was turned back and forth at 37°C until the blood in the tube did not move back and forth.

### Sample Preparation

At least 8 ml of rat urine during the hypercoagulable state was centrifuged at 2,500 g for 30 min at 4°C immediately after collection. The supernatant was collected and centrifuged at 12,000 g for 30 min at 4°C. Three volumes of pre-cold ethanol were added after removing the pellets and precipitated for 2 h at −20°C. Then, urinary protein pellets were dissolved in 25 mM NH_4_HCO_3_ and subjected to quantitation using the Bradford method and determined by SDS-PAGE 4–12% Bis-Tris gels (Invitrogen, Carlsbad, CA, United States). UA buffer (8 M urea in 0.1 M Tris-HCl, pH 8.5) and 25 mM NH_4_HCO_3_ were added after urine samples (about 200 μg) were loaded on the filter unit (Pall Corporation, Washington, NY, United States). Then, proteins were denatured at 50°C for 1 h by the addition of dithiothreitol to the final concentration 4.5 mM and alkylated in the dark for 35 min by the addition of iodoacetamide to the final concentration of 10 mM. The proteins were digested by trypsin with a protein-to-enzyme ratio of 50:1 and incubated at 37°C overnight. The digested peptides were desalted using a 1 ml OASIS HLB cartridge (Waters, Milford, Massachusetts, United States). The eluate was dried via vacuum evaporation (Thermo Fisher Scientific, Bremen, Germany) and stored at −20°C until LC-MS/MS analysis (Hauck et al., 2010).

### LC-MS/MS Analysis

The digested peptides were dissolved in 0.1% formic acid and loaded on a Michrom Peptide Captrap column (MW 0.5–50 kD, 0.5 × 2 mm; Michrom Bioresources, Auburn, CA, United States). The eluent was transferred to a reversed-phase microcapillary column (0.1 × 150 mm, packed with Magic C18, 3 μm, 200Å; Michrom Bioresources, Auburn, CA, United States) by a Waters Ultra-performance EASY-nLC1200 HPLC system. Elution was performed over a gradient of 5–28% buffer B (buffer A: 0.1% formic acid, 99.9% H_**2**_O; buffer B: 0.1% formic acid, 79.9% ACN, 20% H_2_O; flow rate, 0.3 μL/min) for 60 min. The eluted peptides were analyzed using Orbitrap Fusion Lumos MS system (Thermo Fisher Scientific, Bremen, Germany). The MS data were acquired using an ion spray voltage of 2.1 kV, curtain gas of 20 PSI, nebulizer gas of 30 PSI, and an interface heater temperature of 300°C. The precursors were acquired in 500 ms ranging from 350 to 1,550 m*/z* with the resolution set to 120,000, and the product ion scans were acquired in 50 ms ranging from 250 to 1800 m*/z* with the resolution set to 60,000. Dynamic exclusion was employed with a 60-s window to prevent the repetitive selection of the same peptide. Each sample was analyzed at least three times.

### Data Analysis

All MS/MS data in the mascot generic format were analyzed using the Mascot server (version 2.4.1, Matrix Science, London, United Kingdom) in the Swissprot 2013 08 database (taxonomy: *Rattus*; containing 9,354 sequences). Search parameters were set as follows: carbamidomethylation of cysteines was set as a fixed modification and oxidation of methionine and protein N-terminal acetylation were set as variable modifications. Tryptic cleavages at only lysine or arginine with up to two missed cleavage sites were allowed. The precursor mass tolerance was set to 10 ppm, and the fragment mass tolerance was set to 0.02 Da. After the Mascot search, the significance threshold and ion score cutoff were adjusted to 0.05. The false discovery rate for peptides was set a threshold value of 1% when the search result was exported. Peptide identifications were accepted if they could be detected with probability ≥95.0% by the Scaffold local false discovery rate algorithm, and protein identifications were accepted if they could be established with probability greater than 99.0% and contain at least two identified peptides (Nesvizhskii et al., 2003). The determination of the relative abundance of changed urinary proteins was carried out according to the following criteria: fold change >2 for each rat and *p* value < 0.05 (data were analyzed by the *t*-test).

### ELISA

Urinary NGAL levels were measured using the ELISA kit (Boster, Wuhan, China). The OD was measured at 450 nm with a microplate reader (Benchmark Plus, BioRad, Hercules, CA, United States) in triplicate.

### Statistical Analysis

The data were expressed as mean ± standard deviation (x±sd) and analyzed using SPSS version 18.0 software (SPSS Inc., Chicago, IL, United States). *p* < 0.05 was considered significant.

## Results

### Hypercoagulable State Rat Model Induced by ETSL

**FIGURE 2:**
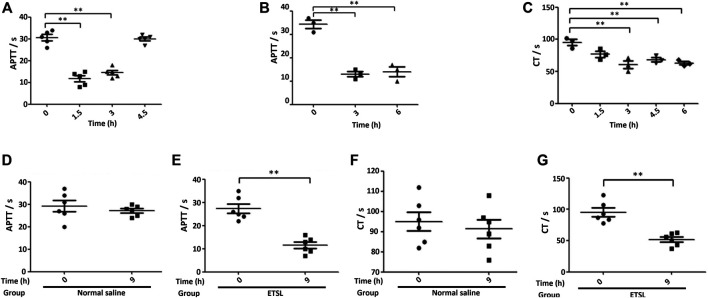
The establishment of ETSL-induced rat model of hypercoagulable state. **(A)**, Changes in the value of APTT **(A)** in rats under single-dose administration (*n* = 10, *p* < 0.05). **(B,** **C)**, APTT **(B)** or CT **(C)** value in rats under second-dose administration (*n* = 10, *p* < 0.05). **(D–G)**, determination of the value of the APTT **(D,E)** or CT **(F,G)** test in different groups of rats 3 h after the third administration, respectively (*n* = 10, *p* < 0.01).

To confirm that we established the hypercoagulable state model, we determined APTT and CT. We found that APTT or CT decreased significantly during the 1.5- to 3-h period (Figure 2). During the 1.5- to 3-h period, there was a plateau period of the hypercoagulable state, which basically returned to normal in 4.5 h. Three ETSL injections were administered, and the hypercoagulable state could be maintained at least for 1.5–9 h, prolonging the time of hypercoagulable state in rats. In addition, ETSL had no fatal effect on the rat model. Therefore, urine collection was controlled during the 1.5- to 9-h period.

### SDS-PAGE Analysis of Urinary Proteins

**FIGURE 3:**
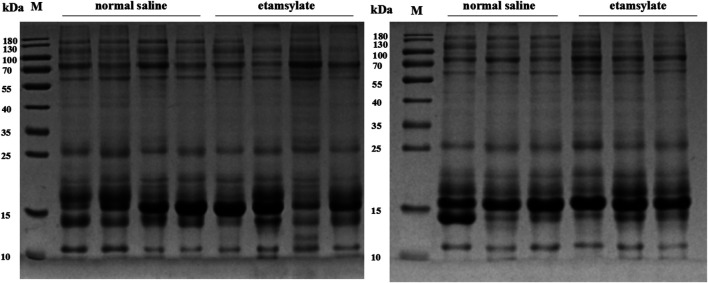
SDS-PAGE analysis of urinary proteins before or after ETSL administration. C1-7, samples from the control group; E1-7, samples from the ETSL administration group.

The effect of ETSL intervention on urine proteins was preliminarily detected by SDS-PAGE analysis. As shown in Figure 3, urine protein strip was intact and the proteins did not degrade. The results of SDS-PAGE showed that there was no significant difference between the hypercoagulable state and the control group, indicating that most of the changed proteins were low-abundance proteins.

### Urine Proteome Analysis of ETSL-Treated Rats

Next, we compared urine proteomic patterns of the control group and model group. A total of 533 proteins were identified including 530 proteins in the control group and 504 proteins in the model group, with 501 proteins in both groups. Among them, 20 proteins with significant differences between two groups were selected (Table 1). Based on the Urinary Protein Biomarkers database (Shao et al., 2011), nine of 20 proteins were candidate biomarkers, such as glypican-3 and L-xylulose reductase.

**TABLE 1:** Dynamic changes in urine proteome in ETSL-induced hypercoagulable state.

Entry	Accession number	Description	Fold change	Mass (Da)	*p* Value	Diseases
Q9QZK9	DNS2B_RAT	Deoxyribonuclease-2-beta	8.33↑	40,472	0.0013	
P07897	PGCA_RAT	Aggrecan core protein	5.6↑	221,118	0.011	
Q62638	GSLG1_RAT	Golgi apparatus protein 1	4.38↑	133,557	0.0027	
B0BNN3	CAH1_RAT	Carbonic anhydrase 1	4.13↑	28,300	0.0008	Non–small cell lung cancer (Wang et al., 2016a)
P13265	GPC3_RAT	Glypican-3	3.33↑	67,049	0.011	Hepatocellular carcinoma (Jia et al., 2015), pancreatic ductal adenocarcinoma (Yao et al., 2016)
O55004	RNAS4_RAT	Ribonuclease 4	3.19↑	16,903	0.0059	
P30152	NGAL_RAT	Neutrophil gelatinase–associated lipocalin	2.55↑	22,476	0.00065	Renal injury (Ozer et al., 2010; Rouse et al., 2011), kidney toxicity (Hoffmann et al., 2010; Tonomura et al., 2010; Fuchs et al., 2012)
Q63678	ZA2G_RAT	Zinc-alpha-2-glycoprotein	1.94↑	34,017	0.018	Diabetic nephropathy (Wang et al., 2016b), colon cancer (Xue et al., 2015)
P15978	HA11_RAT	Class I histocompatibility antigen	1.75↑	36,570	0.00068	
Q6MG61	CLIC1_RAT	Chloride intracellular channel protein 1	1.52↓	26,981	0.035	Ovarian cancer (Tang et al., 2013), intraperitoneal metastasis in serous epithelial ovarian cancer (Ye et al., 2015), malignant-transformed hydatidiform moles (Shi et al., 2011)
P47853	PGS1_RAT	Biglycan	1.75↓	41,706	0.043	Cartilage degradation in osteoarthritis (Barreto et al., 2015), gastric cancer (Wang et al., 2011)
P50399	GDIB_RAT	Rab GDP dissociation inhibitor beta	1.77↓	50,537	0.0083	
P04904	GSTA3_RAT	Glutathione S-transferase alpha-3	1.81↓	25,319	0.0018	
P16617	PGK1_RAT	Phosphoglycerate kinase 1	1.82↓	44,538	0.026	
Q8CG45	ARK72_RAT	Aflatoxin B1 aldehyde reductase member 2	2.1↓	40,675	0.046	
P62959	HINT1_RAT	Histidine triad nucleotide-binding protein 1	2.88↓	13,777	0.014	Type 2 diabetes (Chu et al., 2013)
Q64119	MYL6_RAT	Myosin light polypeptide 6	3.88↓	16,975	0.016	
Q920P0	DCXR_RAT	L-xylulose reductase	7↓	25,720	0.031	Prostate adenocarcinoma (Cho-Vega et al., 2008)
P02651	APOA4_RAT	Apolipoprotein A-IV	7.8↓	44,456	0.00034	Cerebral ischemia (Sironi et al., 2001)
P02091	HBB1_RAT	Hemoglobin subunit beta-1	7.83↓	15,979	0.00052	

Homologous proteins of different species have similar structure and function. To extend our findings of rat proteomics to humans, we searched human homologs of 20 rat proteins by sequence alignment using Uniprot software. Total 13 human proteins were identified, including nine proteins (Golgi apparatus protein 1, carbonic anhydrase 1, glypican-3, chloride intracellular channel protein 1, biglycan, Rab GDP dissociation inhibitor beta, phosphoglycerate kinase 1, L-xylulose reductase, and hemoglobin subunit beta-1) in human core urinary proteome (Table 2).

**TABLE 2:** Corresponding human orthologs of differential proteins in ETSL-induced hypercoagulable state.

Uniprot (rat)	Human ensemble gene ID	Uniprot (human)	Protein name	hCUP	Coagulation-related
Q62638	ENSG00000078369	Q92896–2	Golgi apparatus protein 1	Yes	
B0BNN3	ENSG00000133742	P00915	Carbonic anhydrase 1	Yes	
P13265	ENSG00000147257	P51654	Glypican-3	Yes	Yes
Q6MG61	ENSG00000213719	O00299	Chloride intracellular channel protein 1	Yes	
P47853	ENSG00000182492	P21810	Biglycan	Yes	Yes
P50399	ENSG00000057608	P50395	Rab GDP dissociation inhibitor beta	Yes	
P16617	ENSG00000102144	P00558	Phosphoglycerate kinase 1	Yes	
Q920P0	ENSG00000169738	Q7Z4W1	L-xylulose reductase	Yes	
P02091	ENSG00000244734	P68871	Hemoglobin subunit beta-1	Yes	
O55004	ENSG00000258818	P34096	Ribonuclease 4	No	
Q8CG45	ENSG00000053371	O43488	Aflatoxin B1 aldehyde reductase member 2	No	
P62959	ENSG00000169567	P49773	Histidine triad nucleotide-binding protein 1	No	
Q64119	ENSG00000092841	P60660–2	Myosin light polypeptide 6	No	Yes

hCUP, human core urinary proteome.

### The Characteristics of the Tissue Distribution of Human Orthologs

**FIGURE 4:**
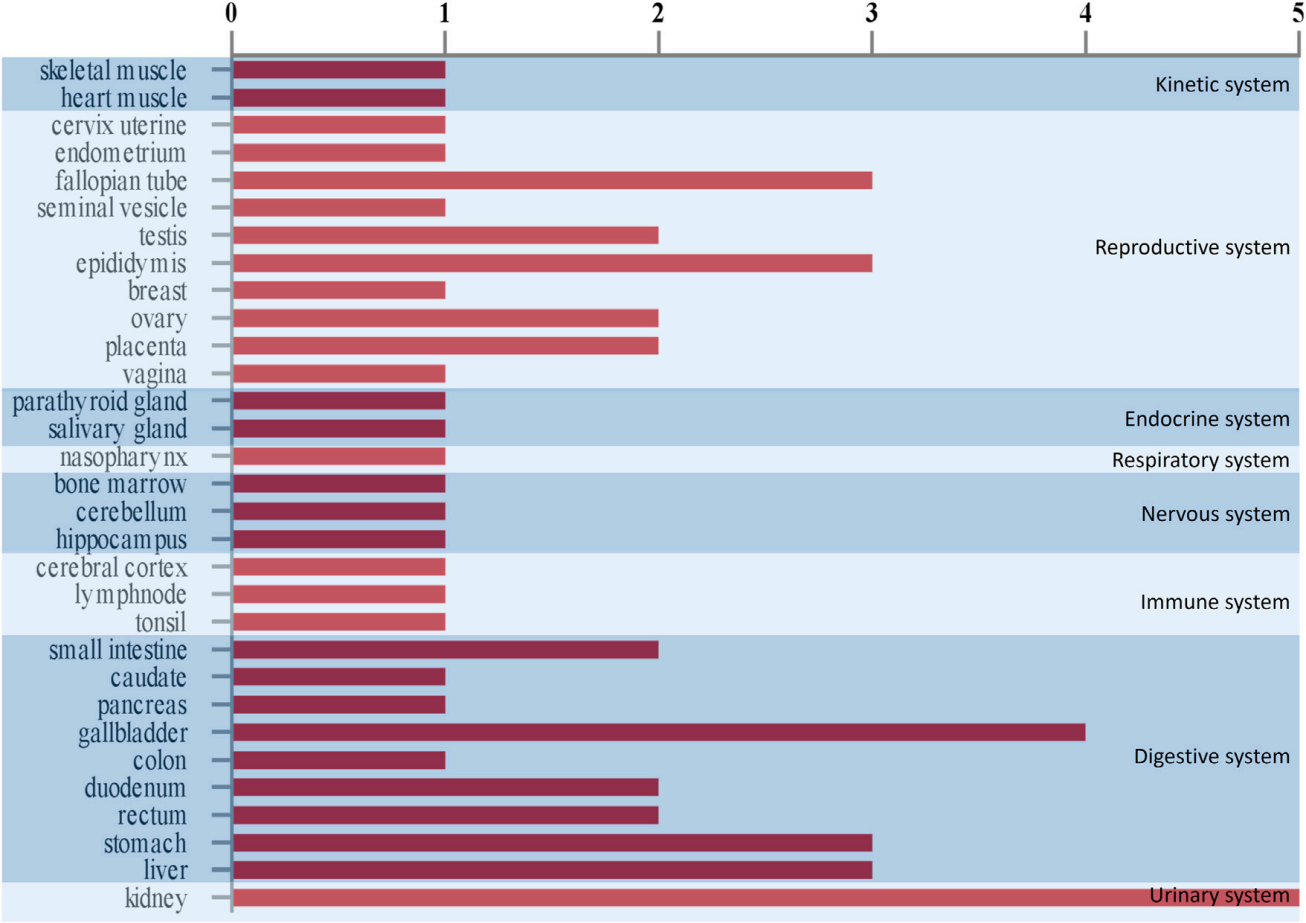
Tissue expression *in vivo* schematic representation of urinary differential proteins in the hypercoagulable state induced by ETSL. The abscissa means the number of proteins that are highly expressed in a tissue. The ordinate means the type of tissues.

Based on the comparison with Human Protein Atlas (http: //www.proteinatlas.org), we found that human homologs of seven differential proteins were highly expressed in 29 tissues in humans (Figure 4). Among these tissues, the kidneys predominated followed by the digestive system and reproductive system. These results suggest that the kidneys are the most responsive organs in the body to the ETSL-induced hypercoagulable state.

### ELISA for NGAL Quantitation in Rat Urine

NGAL belongs to the family of apolipoproteins, and it is covalently linked to neutrophil gelatinase. When renal tubular epithelial cells are stimulated by ischemia and other factors, NGAL can be induced to be expressed in large amounts after 2 h of reperfusion (Zhao et al., 2010).

**FIGURE 5:**
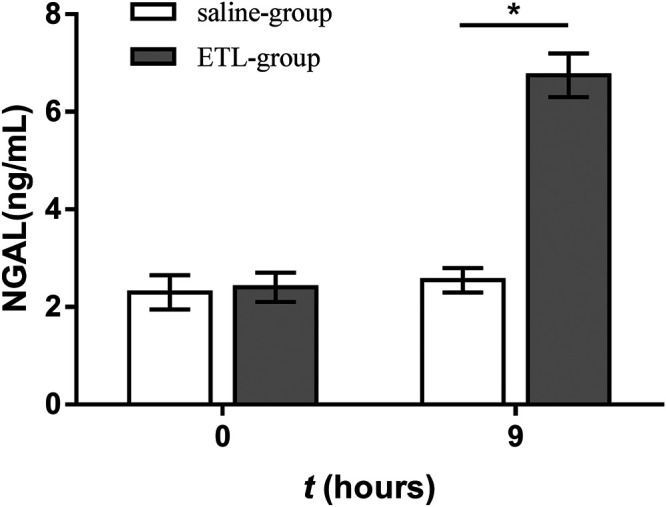
Comparison of urine NGAL concentration in hypercoagulable rats with the control group at corresponding time points (*n* = 3, x ± s). *p < 0.05.

On day 0, NGAL concentration in the urine of the control group and ETSL group showed no significant difference, but NGAL concentration in urine samples of rats treated by ETSL was significantly different from that of the control group during the maintenance period of 1.5–9 h of the hypercoagulable state, showing a significant increase trend (*p* < 0.05, Figure 5). The concentration of NGAL in the urine of rats in the experimental group induced by ETSL was about three times that of the control group.

## Discussion

A variety of diseases such as atherosclerosis, tumor, diabetes, and kidney syndrome are associated with the hypercoagulable state (Piatek et al., 2012; Geddings and Mackman, 2013; Youngwon et al., 2013; Barbosa, 2014; Elyamany et al., 2014; Ahluwalia and Sreedharanunni, 2016). It is generally believed that the occurrence of the hypercoagulable state is due to the coagulation dysfunction. In this study, we employed ETSL to induce the coagulation state in the rat model. Based on the model, for the first time, we systematically investigated dynamic changes of urinary proteome during the acute hypercoagulable state. ETSL is a commonly used clinical hemostatic drug to stop bleeding in surgery, and the main action mechanism is the promotion of platelet adhesion, aggregation and activation, and the release of thrombolytic active substances from platelets to promote vasoconstriction, leading to the activation of coagulation and hemostatic effect. Segura et al. showed that ETSL increased P-selectin expression and leukocyte–platelet aggregate formation both *in vitro* and *in vivo* (Segura et al., 2007). In total, 20 differential proteins with significant changes were detected in urinary proteome; among them, nine proteins were known disease biomarkers and 13 proteins had human counterparts, including nine proteins in human core urinary proteome (Nagaraj and Mann, 2011). These results suggest that human core urinary proteome may change to some extent under the ETSL-induced acute hypercoagulable state.

Human core urinary proteome contains a variety of proteins directly involved in the coagulation: coagulation factors II, V, and XI; kininogen; tissue factor pathway inhibitor; fibrinogen α chain and γ chain; antithrombin III; and thrombin inhibitor. Surprisingly, none of these proteins appeared in the list of nine human homologous proteins we identified.

Based on the urinary proteome analysis, we identified 20 differential proteins with the potential as biomarker of the hypercoagulable state, including five proteins which have been implicated in the regulation of coagulation. Glypican-3 (GPC3) showed a significant positive correlation with alpha-fetoprotein expression, while elevated alpha-fetoprotein level has reported to be associated with portal vein thrombosis (Saito et al., 2008; Jeong et al., 2017). We speculate that elevated GPC3 may be an indicator of the hypercoagulable state in the body which will develop hepatic portal vein thrombosis.

The majority of fetal growth restriction (FGR) cases are associated with placental insufficiency, which can result from placental thrombosis. Reduced biglycan (PGS1) expression may cause placental thrombosis and the pathogenesis of idiopathic FGR (Murthi et al., 2010). In the urine of ETSL-treated rats, we detected a significant decrease in the content of biglycan. This is consistent with the trend of hypercoagulable state. It also suggests that the decrease of biglycan content in urine is not due to the decrease of hemofiltration, but due to the decrease of the expression of biglycan.

Myosin light polypeptide 6 (MYL6) expression decreased in the platelets of patients with deep vein thrombosis (DVT) (Nascimento et al., 2014). The decline of MYL6 level in the urine may be due to reduced release of MYL6. Considering that the main target of ETSL is the platelet, the levels of protein related to platelet function would change during the ETSL-induced hypercoagulable state.

Apolipoprotein A-IV (APOA4) is an endogenous inhibitor of thrombosis (Xu et al., 2014). The absence of APOA4 in the blood enhanced *ex vivo* thrombus growth. In the ETSL-induced hypercoagulable state model, the APOA4 level in urine significantly decreased. This indicates that the content of APOA4, which inhibits thrombus formation, would decrease during the hypercoagulable state. The APOA4 content in blood is very likely to be consistent with that in urine. Therefore, we speculate that the decrease of APOA4 content in urine is due to the decrease of its expression during the hypercoagulable state.

Neutrophil gelatinase-associated lipocalin (NGAL) expression in the plasma increased following deep venous thrombosis of lower extremities (de Franciscis et al., 2016). Also, NAGL expression increased in patients with thrombotic hemorrhoids (Serra et al., 2016). Therefore, increased NAGL expression indicates that the body may be in the hypercoagulable state and prone to thrombosis. In renal ischemic injury, damaged tubular epithelial cells can produce large amounts of NGAL. On one hand, NGAL can induce apoptosis of infiltrating neutrophils in the renal tubule-interstitium to protect the kidney tissue from inflammatory cells; on the other hand, they can induce the transformation of renal mesenchymal cells into renal tubular epithelial cells, thereby inducing the regeneration of renal tubular epithelial cells (Mishra et al., 2003; Mishra et al., 2004). Therefore, NGAL plays a role as a protective factor in acute kidney injury caused by ischemia. NGAL may also act as an iron transporter and induce the generation of heme oxygenase, which is beneficial to the regeneration of injured tubules (Mori et al., 2005). It is generally believed that the NGAL level in urine is positively correlated with the renal tubular injury score of the body. In the hypercoagulable state induced by ETSL, we found an increased NGAL content in urine, but the magnitude of increase was much lower than that induced by acute kidney injury. These results suggest that the ETSL-induced hypercoagulable state has a certain effect on rat kidneys, but this effect is far less intense than acute kidney injury.

Surprisingly, we found that the hypercoagulable state of the body may not involve direct changes in coagulation factors *in vivo* but causes changes in the upstream regulatory points of the coagulation cascade system, consistent with our previous studies (Jing et al., 2019a; Jing et al., 2019b). Upon further stimuli, the coagulation cascade system is triggered and eventually leads to the development of thrombus. Therefore, the protein interaction network in the hypercoagulable state is complex and needs further exploration.

**FIGURE 6:**
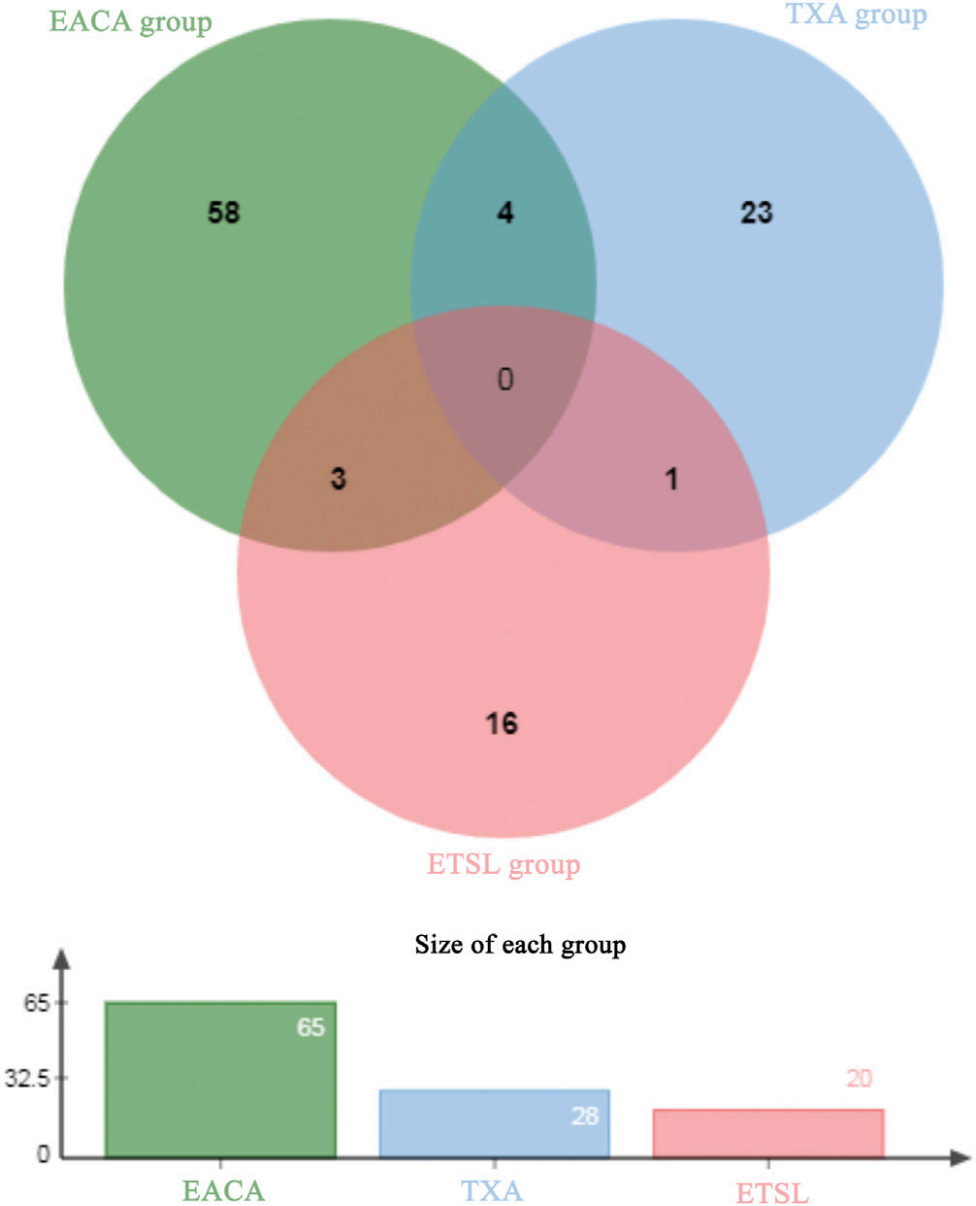
Venn plot of common differential urinary proteins of hypercoagulable states induced by different mechanisms. The Venn diagram is based on the website of jvenn. toulouse.inra.fr/app/example.html.

Interestingly, our results showed that the formation and persistence of hypercoagulable state had different impacts on different tissues and organs. During the hypercoagulable state, changes in protein expression or protein content in the kidney and digestive and reproductive systems were significant. Furthermore, we compared the urinary differential protein profile of the ETSL-induced hypercoagulable state with those of the other two types of drug-induced hypercoagulable states (Jing et al., 2019a; Jing et al., 2019b). As shown in Table 3 and Figure 6, we found no common differential proteins in the acute hypercoagulable state induced by three classes of drugs: ETSL, TXA, or EACA. However, MYL6 protein showed a downward trend in both ETSL and TXA models. DOPD, FAAA, FZD2, and SBP1 proteins showed a downward trend in both TXA and EACA models. NGAL protein showed an upward trend, while GSTA3 and HINT1 proteins showed a downward trend in both ETSL and EACA models. While we found no common urinary differential protein shared by all kinds of acute hypercoagulable states, we identified eight differential proteins shared by different groups and the change trend of these common differential proteins was consistent. These results suggest some common mechanisms or pathways of the hypercoagulable states caused by different factors, although the details of these mechanisms need further investigation such as by expression profiling (Hou et al., 2020). We postulate that these shared differential urine proteins could be used as candidate urinary biomarkers for the hypercoagulable state and can provide new clues for clinical diagnosis of hypercoagulable state.

**TABLE 3:** Common urinary differential proteins shared by different hypercoagulable state models.

Accession	Description	*p* Value tendency	ETSL-group FC	TXA-group FC	EACA-group FC
P30152	Neutrophil gelatinase-associated lipocalin	Up	2.55↑	–	1.54↑
P04904	Glutathione S-transferase alpha-3	Down	1.81↓	–	4.2↓
P62959	Histidine triad nucleotide-binding protein 1	Down	2.88↓	–	8↓
Q64119	Myosin light polypeptide 6	Down	3.88↓	2.5↓	–
P80254	D-dopachrome decarboxylase	Down	–	1.73↓	5.49↓
P25093	Fumarylacetoacetase	Down	–	2.07↓	12.5↓
Q08464	Frizzled-2	Down	–	2.53↓	2.75↓
Q8VIF7	Selenium-binding protein 1	Down	–	4.86↓	17↓

FC, fold change; −, not identified; NGAL, GSTA3 and HINT1, common urinary differential proteins identified between the ETSL group and EACA group; MYL6, common urinary differential proteins identified between the ETSL group and TXA group; DOPD, FAAA, FZD2 and SBP1, common proteins between the TXA group and EACA group, respectively.

In conclusion, we demonstrated that urinary proteome changed during the hypercoagulable state induced by ETSL and identified differential proteins which will help in in-depth understanding of the mechanism of the hypercoagulable state. Further clinical studies are needed to validate differential proteins as candidate biomarkers in early prediction, diagnosis, and prevention of thrombosis-related diseases.

## Data Availability

The original contributions presented in the study are included in the article/Supplementary Material, and further inquiries can be directed to the corresponding author.

## Abbreviations

APTT: activated partial thromboplastin time; CT: clotting time; DOPD: D-dopachrome decarboxylase; DTT: dithiothreitol; EACA: ε-aminocaproic acid; ETSL: etamsylate; FAAA: fumarylacetoacetase; FZD2: Frizzled-2; HINT1: Histidine triad nucleotide-binding protein 1; GSTA3: Glutathione S-transferase alpha-3; LC-MS/MS: liquid chromatography-mass spectrometry/mass spectrometry; MYL6: Myosin light polypeptide 6; NGAL: Neutrophil gelatinase-associated lipocalin; TXA: tranexamic acid; SBP1: Selenium-binding protein 1; SDS-PAGE: sodium dodecyl sulfate polyacrylamide gel electrophoresis; UA: urea; respectively.
