# Supplementary material for: Proteome Analysis of Urinary Biomarkers in Acute Hypercoagulable State Rat Model
Source: Front Mol Biosci. 2021 Apr 30;8:634606. doi: 10.3389/fmolb.2021.634606 (PMC8119894; doi:10.3389/fmolb.2021.634606)
Supplement: Supplementary file 1 [file Table1.DOCX]

Supplementary Table 1. Identification of urinary proteins in hypercoagulable state rat model induced by etamsylate

| Accession Number | Molecular Weight（kDa） | Spectral Counts | | | | | | | | | | | |
| --- | --- | --- | --- | --- | --- | --- | --- | --- | --- | --- | --- | --- | --- |
|  |  | NS 1_1 | NS 1_2 | NS 2_1 | NS 2_2 | NS 3_1 | NS 3_2 | ES 1_1 | ES 1_2 | ES 2_1 | ES 2_2 | ES 3_1 | ES 3_2 |
| AMPN_RAT | 109 | 80 | 78 | 61 | 57 | 78 | 75 | 65 | 64 | 56 | 59 | 46 | 46 |
| ACTG_RAT [3] | 42 | 51 | 57 | 48 | 51 | 65 | 60 | 38 | 45 | 39 | 49 | 39 | 36 |
| A1AT_RAT | 46 | 29 | 29 | 33 | 33 | 26 | 30 | 24 | 27 | 24 | 27 | 26 | 30 |
| DPP4_RAT | 88 | 33 | 36 | 24 | 25 | 32 | 30 | 26 | 27 | 21 | 25 | 22 | 21 |
| FETUA_RAT | 38 | 25 | 19 | 31 | 27 | 17 | 23 | 15 | 16 | 16 | 21 | 18 | 17 |
| GGT1_RAT | 62 | 26 | 28 | 21 | 24 | 25 | 23 | 26 | 18 | 23 | 18 | 16 | 16 |
| CBPQ_RAT | 52 | 19 | 25 | 23 | 25 | 19 | 16 | 17 | 22 | 15 | 14 | 14 | 16 |
| AK1A1_RAT | 37 | 13 | 12 | 13 | 13 | 15 | 12 | 11 | 9 | 7 | 8 | 10 | 8 |
| TPIS_RAT | 27 | 11 | 13 | 13 | 12 | 15 | 14 | 5 | 9 | 8 | 8 | 8 | 8 |
| ALDOB_RAT | 40 | 13 | 10 | 10 | 9 | 16 | 8 | 10 | 9 | 4 | 5 | 4 | 9 |
| K1C10_RAT | 57 | 15 | 17 | 13 | 11 | 10 | 10 | 4 | 3 | 6 | 7 | 1 | 2 |
| CNDP2_RAT | 53 | 13 | 13 | 9 | 4 | 14 | 9 | 6 | 6 | 6 | 8 | 1 | 4 |
| GDIB_RAT | 51 | 7 | 14 | 8 | 10 | 14 | 15 | 5 | 8 | 7 | 5 | 8 | 4 |
| K2C1_RAT | 65 | 10 | 17 | 11 | 9 | 12 | 9 | 1 | 1 | 7 | 2 | 1 | 0 |
| PGS1_RAT | 42 | 7 | 6 | 11 | 12 | 7 | 8 | 4 | 8 | 1 | 4 | 8 | 6 |
| ASSY_RAT | 46 | 9 | 9 | 4 | 2 | 8 | 7 | 1 | 4 | 5 | 4 | 4 | 4 |
| S1C2A_RAT [2] | 35 | 8 | 8 | 3 | 3 | 10 | 8 | 1 | 0 | 3 | 5 | 1 | 3 |
| CLIC1_RAT | 27 | 6 | 7 | 6 | 5 | 10 | 9 | 5 | 3 | 8 | 2 | 5 | 4 |
| RL40_RAT (+3) | 15 | 6 | 6 | 6 | 8 | 5 | 5 | 5 | 4 | 4 | 6 | 3 | 4 |
| CGL_RAT | 44 | 7 | 8 | 3 | 4 | 8 | 10 | 6 | 4 | 5 | 2 | 2 | 1 |
| PGK1_RAT | 45 | 9 | 6 | 7 | 4 | 8 | 5 | 2 | 2 | 5 | 4 | 3 | 6 |
| ACOC_RAT | 98 | 8 | 6 | 3 | 2 | 6 | 6 | 2 | 1 | 5 | 2 | 4 | 0 |
| GSTA3_RAT | 25 | 6 | 6 | 4 | 5 | 5 | 6 | 5 | 3 | 2 | 3 | 1 | 3 |
| VATB2_RAT | 57 | 7 | 6 | 2 | 3 | 5 | 5 | 1 | 2 | 3 | 3 | 3 | 1 |
| 3HAO_RAT | 33 | 6 | 6 | 3 | 3 | 3 | 4 | 5 | 2 | 3 | 1 | 0 | 1 |
| MYO1C_RAT | 120 | 6 | 6 | 2 | 1 | 7 | 6 | 1 | 3 | 3 | 3 | 1 | 1 |
| APOA4_RAT | 44 | 5 | 4 | 7 | 4 | 4 | 5 | 0 | 0 | 0 | 0 | 3 | 2 |
| K22E_RAT | 69 | 1 | 2 | 8 | 10 | 7 | 10 | 0 | 0 | 0 | 0 | 0 | 0 |
| PROS_RAT | 75 | 4 | 3 | 2 | 1 | 3 | 3 | 0 | 1 | 0 | 3 | 1 | 1 |
| ARK72_RAT | 41 | 4 | 2 | 3 | 1 | 4 | 3 | 1 | 1 | 1 | 2 | 3 | 0 |
| MGLL_RAT | 33 | 3 | 4 | 2 | 1 | 4 | 4 | 0 | 2 | 2 | 1 | 2 | 1 |
| S10A8_RAT | 10 | 4 | 4 | 3 | 4 | 1 | 2 | 0 | 1 | 0 | 1 | 2 | 3 |
| S10A9_RAT | 13 | 3 | 5 | 3 | 4 | 1 | 1 | 0 | 1 | 0 | 0 | 2 | 0 |
| VDAC1_RAT | 31 | 3 | 4 | 1 | 0 | 3 | 6 | 1 | 1 | 0 | 0 | 0 | 0 |
| HBB1_RAT | 16 | 4 | 4 | 1 | 2 | 4 | 3 | 1 | 1 | 0 | 0 | 0 | 0 |
| SNP23_RAT | 23 | 2 | 2 | 1 | 1 | 2 | 2 | 0 | 1 | 2 | 0 | 1 | 0 |
| K2C6A_RAT | 59 | 4 | 2 | 2 | 1 | 2 | 1 | 1 | 0 | 1 | 1 | 1 | 1 |
| TPMT_RAT | 28 | 4 | 4 | 0 | 0 | 2 | 2 | 0 | 1 | 0 | 0 | 0 | 0 |
| MYL6_RAT | 17 | 3 | 2 | 2 | 2 | 0 | 2 | 0 | 1 | 1 | 0 | 0 | 1 |
| 6PGD_RAT | 53 | 3 | 2 | 1 | 1 | 2 | 2 | 1 | 0 | 1 | 0 | 1 | 1 |
| HINT1_RAT | 14 | 2 | 1 | 1 | 1 | 2 | 2 | 1 | 0 | 1 | 0 | 1 | 0 |
| WDR1_RAT | 66 | 2 | 3 | 1 | 0 | 2 | 2 | 1 | 0 | 0 | 0 | 0 | 0 |
| DPP3_RAT | 83 | 2 | 2 | 0 | 0 | 2 | 1 | 0 | 0 | 0 | 0 | 0 | 0 |
| NIT1_RAT | 32 | 1 | 2 | 1 | 1 | 1 | 2 | 0 | 0 | 1 | 1 | 1 | 1 |
| S47A1_RAT | 61 | 2 | 1 | 1 | 1 | 1 | 2 | 0 | 0 | 1 | 0 | 1 | 1 |
| GSTA4_RAT | 26 | 2 | 3 | 0 | 1 | 2 | 1 | 0 | 0 | 0 | 1 | 0 | 0 |
| COMT_RAT | 30 | 0 | 1 | 1 | 3 | 2 | 1 | 0 | 0 | 0 | 0 | 0 | 0 |
| NDRG1_RAT | 43 | 1 | 1 | 0 | 1 | 2 | 3 | 0 | 0 | 0 | 0 | 0 | 1 |
| DCXR_RAT | 26 | 2 | 1 | 1 | 1 | 3 | 0 | 1 | 0 | 0 | 0 | 0 | 0 |
| K1C40_RAT | 48 | 2 | 2 | 0 | 0 | 1 | 2 | 0 | 0 | 0 | 0 | 0 | 0 |
| MDR1_RAT | 141 | 2 | 2 | 0 | 0 | 1 | 2 | 0 | 0 | 0 | 0 | 0 | 0 |
| ACOT1_RAT | 46 | 1 | 2 | 0 | 0 | 1 | 2 | 0 | 0 | 0 | 0 | 0 | 0 |
| DMBT1_RAT | 156 | 0 | 1 | 2 | 1 | 1 | 1 | 0 | 0 | 0 | 0 | 0 | 0 |
| GIPC2_RAT | 34 | 3 | 2 | 0 | 0 | 1 | 1 | 0 | 0 | 0 | 0 | 0 | 0 |
| RAC1_RAT | 21 | 1 | 2 | 0 | 0 | 2 | 1 | 0 | 0 | 0 | 0 | 0 | 0 |
| SC5A6_RAT | 69 | 1 | 2 | 0 | 0 | 1 | 1 | 0 | 0 | 0 | 0 | 0 | 0 |
| KLK1_RAT | 29 | 22 | 23 | 33 | 27 | 25 | 26 | 26 | 33 | 33 | 27 | 34 | 35 |
| AMYP_RAT | 57 | 25 | 25 | 24 | 27 | 24 | 26 | 39 | 35 | 26 | 28 | 28 | 32 |
| SAMP_RAT | 26 | 16 | 16 | 23 | 23 | 17 | 17 | 23 | 30 | 22 | 19 | 25 | 28 |
| HPT_RAT | 39 | 16 | 16 | 12 | 14 | 8 | 9 | 13 | 19 | 16 | 16 | 21 | 19 |
| NPTN_RAT | 44 | 9 | 12 | 12 | 10 | 11 | 9 | 16 | 17 | 12 | 10 | 15 | 13 |
| BTD_RAT | 58 | 10 | 10 | 13 | 13 | 10 | 13 | 11 | 16 | 15 | 15 | 18 | 15 |
| GILT_RAT | 28 | 12 | 11 | 11 | 12 | 12 | 12 | 13 | 14 | 14 | 14 | 13 | 10 |
| DIAC_RAT | 42 | 8 | 12 | 11 | 8 | 13 | 12 | 16 | 15 | 12 | 14 | 11 | 13 |
| KLK6_RAT | 29 | 9 | 9 | 7 | 7 | 17 | 16 | 13 | 13 | 18 | 16 | 23 | 25 |
| PEBP1_RAT | 21 | 3 | 3 | 3 | 4 | 7 | 7 | 4 | 5 | 13 | 14 | 17 | 19 |
| NUCB1_RAT | 54 | 4 | 3 | 12 | 13 | 3 | 4 | 10 | 12 | 15 | 11 | 14 | 10 |
| PTGDS_RAT | 21 | 4 | 5 | 6 | 4 | 7 | 4 | 5 | 8 | 7 | 7 | 6 | 8 |
| DNS2B_RAT | 40 | 0 | 0 | 0 | 0 | 7 | 5 | 16 | 19 | 12 | 7 | 12 | 7 |
| TTHY_RAT | 16 | 6 | 6 | 6 | 8 | 6 | 5 | 6 | 8 | 8 | 6 | 8 | 9 |
| NID1_RAT | 36 | 6 | 5 | 9 | 3 | 6 | 4 | 7 | 9 | 6 | 7 | 10 | 10 |
| HA11_RAT | 37 | 4 | 7 | 9 | 9 | 9 | 8 | 16 | 12 | 14 | 10 | 15 | 13 |
| SVS2_RAT | 46 | 0 | 0 | 1 | 2 | 2 | 2 | 16 | 15 | 12 | 7 | 10 | 7 |
| HEXB_RAT | 62 | 3 | 2 | 4 | 2 | 2 | 3 | 6 | 5 | 7 | 7 | 3 | 3 |
| OSTP_RAT | 35 | 3 | 3 | 1 | 4 | 4 | 4 | 2 | 3 | 7 | 7 | 10 | 10 |
| NGAL_RAT | 22 | 1 | 3 | 3 | 2 | 2 | 1 | 4 | 4 | 7 | 5 | 6 | 6 |
| ZA2G_RAT | 34 | 2 | 2 | 1 | 3 | 1 | 5 | 5 | 3 | 5 | 3 | 5 | 6 |
| RNAS4_RAT | 17 | 0 | 2 | 2 | 1 | 2 | 1 | 2 | 3 | 4 | 4 | 8 | 6 |
| GSLG1_RAT | 134 | 0 | 1 | 0 | 2 | 2 | 0 | 4 | 5 | 2 | 2 | 4 | 5 |
| CATS_RAT | 37 | 2 | 1 | 2 | 1 | 3 | 2 | 2 | 3 | 5 | 3 | 4 | 2 |
| PGCA_RAT | 221 | 0 | 1 | 0 | 0 | 0 | 1 | 1 | 2 | 0 | 3 | 3 | 2 |
| GPC3_RAT | 67 | 2 | 0 | 1 | 1 | 0 | 0 | 2 | 1 | 2 | 5 | 2 | 3 |
| CAH1_RAT | 28 | 1 | 1 | 0 | 0 | 0 | 1 | 2 | 2 | 3 | 2 | 2 | 1 |
| PRVA_RAT | 12 | 2 | 1 | 2 | 1 | 0 | 0 | 2 | 2 | 3 | 2 | 2 | 1 |
| MYG_RAT | 17 | 0 | 0 | 1 | 0 | 0 | 0 | 2 | 4 | 1 | 0 | 1 | 1 |
| NPT2A_RAT | 69 | 0 | 0 | 0 | 0 | 0 | 1 | 0 | 1 | 1 | 1 | 2 | 1 |
| 8ODP_RAT | 18 | 0 | 0 | 0 | 1 | 0 | 0 | 1 | 2 | 0 | 1 | 1 | 3 |
| MUP_RAT [2] | 21 | 260 | 249 | 362 | 363 | 269 | 266 | 365 | 364 | 299 | 357 | 305 | 341 |
| ALBU_RAT | 69 | 237 | 225 | 220 | 202 | 204 | 222 | 237 | 230 | 175 | 203 | 190 | 224 |
| LRP2_RAT | 519 | 142 | 137 | 130 | 129 | 112 | 108 | 145 | 124 | 125 | 105 | 118 | 100 |
| UROM_RAT | 71 | 125 | 122 | 127 | 119 | 109 | 114 | 159 | 154 | 95 | 98 | 113 | 115 |
| EGF_RAT | 124 | 121 | 128 | 141 | 155 | 109 | 116 | 123 | 128 | 115 | 127 | 117 | 121 |
| A1M_RAT | 167 | 100 | 109 | 123 | 134 | 99 | 107 | 124 | 122 | 102 | 99 | 116 | 108 |
| UP2_RAT | 11 | 73 | 73 | 83 | 88 | 59 | 56 | 54 | 59 | 104 | 128 | 79 | 107 |
| A1I3_RAT [2] | 164 | 77 | 67 | 84 | 79 | 56 | 54 | 105 | 100 | 68 | 70 | 70 | 59 |
| KLK9_RAT [2] | 28 | 56 | 63 | 63 | 67 | 88 | 89 | 82 | 70 | 65 | 79 | 80 | 78 |
| TRFE_RAT | 76 | 60 | 66 | 72 | 76 | 67 | 69 | 81 | 74 | 61 | 58 | 69 | 71 |
| HEMO_RAT | 51 | 52 | 47 | 68 | 71 | 59 | 59 | 83 | 64 | 69 | 63 | 66 | 73 |
| SPA3L_RAT | 46 | 88 | 87 | 96 | 104 | 70 | 80 | 78 | 91 | 78 | 94 | 79 | 89 |
| SLC31_RAT | 79 | 69 | 76 | 45 | 35 | 71 | 71 | 48 | 52 | 56 | 56 | 53 | 53 |
| TGM4_RAT | 76 | 49 | 50 | 63 | 58 | 47 | 47 | 56 | 53 | 75 | 70 | 45 | 46 |
| 22P1_RAT | 21 | 55 | 52 | 24 | 34 | 32 | 32 | 43 | 37 | 61 | 69 | 45 | 47 |
| FINC_RAT | 273 | 50 | 51 | 66 | 59 | 45 | 45 | 41 | 47 | 47 | 49 | 53 | 50 |
| PLMN_RAT | 91 | 42 | 48 | 50 | 54 | 37 | 41 | 48 | 45 | 48 | 45 | 50 | 49 |
| MEP1A_RAT | 85 | 44 | 46 | 48 | 50 | 44 | 48 | 55 | 39 | 51 | 36 | 45 | 35 |
| UP1_RAT | 11 | 54 | 55 | 48 | 60 | 27 | 35 | 22 | 28 | 62 | 82 | 26 | 36 |
| AFAM_RAT | 69 | 41 | 45 | 43 | 41 | 33 | 37 | 45 | 38 | 50 | 35 | 46 | 38 |
| GELS_RAT | 86 | 31 | 33 | 35 | 37 | 33 | 34 | 38 | 32 | 31 | 34 | 36 | 40 |
| BGLR_RAT | 75 | 52 | 48 | 52 | 58 | 7 | 6 | 28 | 21 | 39 | 35 | 57 | 50 |
| PIGR_RAT | 85 | 34 | 37 | 37 | 34 | 33 | 31 | 41 | 37 | 37 | 34 | 39 | 33 |
| QSOX1_RAT | 82 | 26 | 24 | 26 | 27 | 41 | 36 | 73 | 74 | 30 | 22 | 24 | 25 |
| CADH1_RAT | 99 | 29 | 30 | 40 | 29 | 35 | 37 | 31 | 36 | 32 | 31 | 32 | 38 |
| SPA3K_RAT | 47 | 55 | 52 | 68 | 65 | 42 | 53 | 48 | 67 | 67 | 65 | 60 | 62 |
| NEP_RAT | 86 | 42 | 38 | 29 | 26 | 52 | 48 | 39 | 25 | 43 | 35 | 39 | 31 |
| AMPE_RAT | 108 | 43 | 39 | 32 | 30 | 35 | 26 | 32 | 29 | 27 | 30 | 31 | 29 |
| AMBP_RAT | 39 | 22 | 24 | 35 | 42 | 30 | 30 | 22 | 28 | 40 | 28 | 37 | 46 |
| DNAS1_RAT | 32 | 26 | 25 | 31 | 28 | 27 | 26 | 41 | 39 | 26 | 30 | 32 | 29 |
| VTDB_RAT | 54 | 23 | 20 | 30 | 34 | 34 | 34 | 24 | 31 | 30 | 35 | 30 | 35 |
| MXRA8_RAT | 43 | 27 | 27 | 31 | 29 | 28 | 27 | 28 | 28 | 26 | 22 | 33 | 27 |
| PSC1_RAT | 13 | 29 | 28 | 26 | 21 | 38 | 33 | 20 | 13 | 36 | 32 | 35 | 34 |
| UP3_RAT | 11 | 28 | 27 | 28 | 37 | 20 | 21 | 11 | 19 | 42 | 63 | 19 | 35 |
| SG2A2_RAT | 11 | 20 | 18 | 9 | 11 | 20 | 34 | 6 | 12 | 22 | 38 | 19 | 32 |
| 22P2_RAT | 21 | 25 | 22 | 19 | 18 | 33 | 32 | 26 | 23 | 35 | 28 | 35 | 31 |
| MEP1B_RAT | 79 | 29 | 27 | 26 | 29 | 33 | 32 | 29 | 25 | 30 | 27 | 18 | 24 |
| CO3_RAT | 186 | 18 | 17 | 31 | 21 | 22 | 23 | 20 | 26 | 21 | 19 | 25 | 27 |
| GSH1_RAT | 73 | 31 | 36 | 17 | 17 | 33 | 31 | 26 | 23 | 24 | 21 | 21 | 16 |
| UROK_RAT | 48 | 19 | 19 | 23 | 21 | 24 | 18 | 35 | 24 | 27 | 25 | 19 | 22 |
| REG3G_RAT | 19 | 14 | 21 | 35 | 36 | 14 | 22 | 15 | 17 | 23 | 21 | 32 | 31 |
| EZRI_RAT | 69 | 34 | 35 | 23 | 27 | 41 | 38 | 33 | 35 | 28 | 27 | 35 | 31 |
| MOES_RAT | 68 | 40 | 37 | 30 | 24 | 41 | 38 | 34 | 28 | 29 | 28 | 28 | 18 |
| KNT1_RAT [2] | 48 | 21 | 24 | 26 | 18 | 21 | 19 | 26 | 26 | 19 | 20 | 23 | 18 |
| CATB_RAT | 37 | 15 | 20 | 25 | 24 | 20 | 14 | 27 | 20 | 27 | 21 | 24 | 23 |
| SPBP_RAT | 31 | 17 | 19 | 12 | 10 | 23 | 23 | 15 | 9 | 34 | 25 | 33 | 29 |
| ENOA_RAT | 47 | 19 | 17 | 17 | 20 | 22 | 19 | 16 | 15 | 19 | 20 | 17 | 20 |
| CFAD_RAT | 28 | 20 | 17 | 20 | 22 | 20 | 17 | 18 | 18 | 28 | 24 | 24 | 21 |
| EST1C_RAT | 60 | 16 | 17 | 17 | 20 | 20 | 19 | 28 | 22 | 18 | 20 | 18 | 18 |
| LYAG_RAT | 106 | 17 | 17 | 20 | 25 | 12 | 18 | 21 | 22 | 11 | 12 | 21 | 18 |
| CBG_RAT | 45 | 14 | 15 | 24 | 22 | 11 | 13 | 27 | 24 | 11 | 13 | 21 | 22 |
| LAC2_RAT | 11 | 16 | 14 | 19 | 26 | 15 | 18 | 15 | 20 | 17 | 21 | 18 | 18 |
| NHRF3_RAT | 57 | 29 | 26 | 12 | 8 | 28 | 22 | 13 | 6 | 16 | 14 | 18 | 17 |
| DPP2_RAT | 55 | 18 | 17 | 19 | 17 | 16 | 17 | 15 | 16 | 16 | 13 | 20 | 15 |
| CATD_RAT | 45 | 18 | 18 | 25 | 24 | 15 | 11 | 23 | 23 | 17 | 11 | 18 | 14 |
| KACB_RAT | 12 | 48 | 50 | 81 | 88 | 77 | 86 | 45 | 65 | 51 | 83 | 84 | 88 |
| CRP_RAT | 25 | 17 | 14 | 12 | 15 | 15 | 13 | 17 | 32 | 19 | 21 | 11 | 13 |
| CERU_RAT | 121 | 19 | 18 | 18 | 16 | 16 | 16 | 24 | 22 | 11 | 13 | 16 | 18 |
| CYTC_RAT | 15 | 17 | 17 | 18 | 20 | 12 | 11 | 12 | 18 | 22 | 21 | 19 | 17 |
| GGH_RAT | 36 | 13 | 16 | 14 | 15 | 14 | 13 | 17 | 12 | 15 | 13 | 15 | 13 |
| CD48_RAT | 28 | 18 | 17 | 21 | 20 | 17 | 12 | 20 | 17 | 14 | 14 | 16 | 14 |
| SMR1_RAT | 16 | 17 | 20 | 12 | 16 | 16 | 12 | 12 | 15 | 16 | 14 | 10 | 13 |
| FETUB_RAT | 42 | 14 | 10 | 18 | 20 | 19 | 20 | 13 | 15 | 12 | 12 | 21 | 19 |
| CUBN_RAT | 399 | 20 | 19 | 13 | 16 | 10 | 12 | 13 | 19 | 11 | 8 | 13 | 13 |
| LG3BP_RAT | 64 | 18 | 18 | 12 | 13 | 15 | 12 | 26 | 14 | 17 | 11 | 17 | 13 |
| CLUS_RAT | 51 | 10 | 12 | 14 | 14 | 16 | 17 | 13 | 15 | 16 | 20 | 18 | 19 |
| PBAS_RAT | 21 | 13 | 18 | 9 | 8 | 14 | 12 | 24 | 24 | 13 | 15 | 11 | 15 |
| FBLN3_RAT | 55 | 12 | 16 | 18 | 15 | 16 | 13 | 20 | 16 | 14 | 13 | 17 | 15 |
| CSPG4_RAT | 252 | 15 | 9 | 21 | 16 | 23 | 16 | 20 | 13 | 8 | 7 | 15 | 15 |
| HSP7C_RAT | 71 | 18 | 20 | 11 | 11 | 16 | 23 | 16 | 14 | 13 | 15 | 11 | 15 |
| NHRF1_RAT | 39 | 15 | 20 | 10 | 10 | 18 | 18 | 16 | 13 | 12 | 12 | 9 | 11 |
| RISC_RAT | 51 | 16 | 15 | 18 | 15 | 8 | 6 | 12 | 13 | 6 | 10 | 8 | 9 |
| KACA_RAT | 12 | 58 | 61 | 59 | 62 | 52 | 75 | 63 | 84 | 64 | 95 | 61 | 69 |
| PSC2_RAT | 13 | 9 | 9 | 10 | 8 | 15 | 10 | 7 | 6 | 12 | 16 | 10 | 15 |
| APOE_RAT | 36 | 9 | 10 | 15 | 12 | 11 | 9 | 16 | 13 | 13 | 8 | 11 | 9 |
| ANGT_RAT | 52 | 12 | 13 | 12 | 11 | 12 | 11 | 12 | 10 | 14 | 10 | 12 | 8 |
| THRB_RAT | 70 | 10 | 9 | 14 | 15 | 14 | 14 | 6 | 5 | 13 | 12 | 17 | 18 |
| G3P_RAT | 36 | 18 | 16 | 11 | 13 | 18 | 15 | 10 | 11 | 15 | 13 | 14 | 12 |
| 6PGL_RAT | 27 | 15 | 13 | 20 | 21 | 7 | 11 | 11 | 11 | 11 | 6 | 14 | 9 |
| A1AG_RAT | 24 | 8 | 6 | 4 | 12 | 9 | 9 | 9 | 13 | 13 | 10 | 15 | 9 |
| ACY1A_RAT | 46 | 9 | 11 | 8 | 10 | 11 | 12 | 9 | 11 | 11 | 8 | 9 | 11 |
| PPAL_RAT | 48 | 14 | 13 | 17 | 14 | 10 | 9 | 15 | 10 | 9 | 5 | 9 | 4 |
| CATC_RAT | 52 | 9 | 6 | 13 | 13 | 8 | 10 | 12 | 10 | 13 | 16 | 10 | 14 |
| SAP_RAT | 61 | 13 | 10 | 15 | 16 | 7 | 7 | 7 | 8 | 14 | 11 | 16 | 10 |
| SODC_RAT | 16 | 11 | 7 | 13 | 14 | 10 | 9 | 12 | 7 | 16 | 13 | 12 | 10 |
| GRN_RAT | 63 | 11 | 11 | 9 | 12 | 11 | 10 | 11 | 11 | 12 | 12 | 11 | 12 |
| B2MG_RAT | 14 | 10 | 7 | 9 | 13 | 9 | 12 | 13 | 13 | 8 | 11 | 7 | 10 |
| ESAM_RAT | 42 | 9 | 8 | 14 | 10 | 10 | 8 | 10 | 13 | 10 | 9 | 11 | 10 |
| LDHB_RAT | 37 | 12 | 9 | 12 | 11 | 8 | 8 | 7 | 11 | 8 | 12 | 13 | 11 |
| AADAT_RAT | 48 | 12 | 16 | 8 | 9 | 15 | 13 | 11 | 10 | 12 | 8 | 4 | 6 |
| ABHEB_RAT | 23 | 11 | 8 | 11 | 9 | 11 | 7 | 12 | 13 | 10 | 10 | 6 | 13 |
| MANBA_RAT | 101 | 12 | 10 | 9 | 11 | 10 | 14 | 10 | 10 | 12 | 5 | 11 | 8 |
| MINP1_RAT | 55 | 9 | 9 | 7 | 11 | 7 | 9 | 7 | 10 | 14 | 9 | 10 | 10 |
| IL4RA_RAT | 87 | 8 | 10 | 11 | 11 | 7 | 10 | 7 | 9 | 10 | 13 | 9 | 12 |
| SPA3N_RAT | 47 | 4 | 7 | 13 | 8 | 9 | 9 | 12 | 12 | 10 | 8 | 14 | 15 |
| PDC6I_RAT | 97 | 13 | 15 | 6 | 3 | 11 | 6 | 12 | 11 | 4 | 8 | 6 | 9 |
| 1433Z_RAT [3] | 28 | 11 | 7 | 9 | 7 | 13 | 10 | 6 | 10 | 9 | 10 | 9 | 8 |
| F16P1_RAT | 40 | 13 | 12 | 4 | 5 | 11 | 16 | 9 | 5 | 7 | 8 | 6 | 8 |
| JAM1_RAT | 32 | 9 | 6 | 8 | 10 | 8 | 7 | 9 | 6 | 13 | 9 | 13 | 12 |
| LEG5_RAT [2] | 16 | 10 | 8 | 11 | 11 | 6 | 6 | 10 | 6 | 7 | 7 | 4 | 6 |
| APOH_RAT | 33 | 7 | 7 | 11 | 10 | 10 | 7 | 6 | 10 | 10 | 7 | 9 | 10 |
| ARSB_RAT | 59 | 6 | 8 | 9 | 9 | 7 | 6 | 9 | 7 | 7 | 5 | 6 | 6 |
| GSH0_RAT | 31 | 7 | 9 | 6 | 8 | 13 | 6 | 12 | 11 | 8 | 7 | 8 | 4 |
| SODE_RAT | 27 | 6 | 8 | 9 | 12 | 7 | 6 | 7 | 8 | 13 | 6 | 11 | 9 |
| TKFC_RAT | 59 | 15 | 11 | 3 | 4 | 12 | 9 | 7 | 6 | 7 | 3 | 5 | 5 |
| CAH2_RAT | 29 | 8 | 6 | 8 | 9 | 7 | 8 | 11 | 10 | 12 | 9 | 7 | 6 |
| LCN5_RAT | 21 | 4 | 4 | 1 | 3 | 13 | 11 | 0 | 0 | 16 | 19 | 9 | 12 |
| PPIA_RAT | 18 | 7 | 6 | 6 | 9 | 7 | 6 | 2 | 12 | 5 | 8 | 7 | 8 |
| WFDC2_RAT | 17 | 6 | 6 | 13 | 9 | 8 | 7 | 9 | 9 | 7 | 9 | 9 | 9 |
| CDHR5_RAT | 91 | 10 | 8 | 7 | 11 | 5 | 7 | 7 | 9 | 5 | 6 | 8 | 6 |
| MDHC_RAT | 36 | 9 | 9 | 9 | 10 | 8 | 10 | 4 | 9 | 7 | 7 | 8 | 9 |
| EF1A1_RAT | 50 | 13 | 9 | 6 | 2 | 11 | 9 | 9 | 8 | 8 | 6 | 5 | 4 |
| ATRN_RAT | 159 | 6 | 7 | 6 | 10 | 5 | 5 | 10 | 9 | 8 | 6 | 7 | 5 |
| CALB1_RAT | 30 | 6 | 6 | 12 | 13 | 4 | 8 | 1 | 0 | 10 | 8 | 9 | 6 |
| CLIC4_RAT | 29 | 6 | 8 | 7 | 7 | 7 | 9 | 7 | 3 | 7 | 8 | 5 | 5 |
| RGN_RAT | 33 | 10 | 8 | 4 | 2 | 7 | 10 | 6 | 7 | 6 | 6 | 4 | 5 |
| CATL1_RAT | 38 | 7 | 6 | 7 | 7 | 8 | 6 | 10 | 7 | 6 | 7 | 6 | 7 |
| CO4_RAT | 192 | 9 | 9 | 4 | 5 | 4 | 6 | 2 | 6 | 3 | 3 | 10 | 9 |
| CRIS1_RAT | 28 | 1 | 2 | 1 | 0 | 11 | 8 | 1 | 1 | 18 | 19 | 13 | 12 |
| EST5A_RAT | 64 | 3 | 2 | 1 | 0 | 7 | 9 | 0 | 0 | 17 | 18 | 8 | 10 |
| TPP1_RAT | 61 | 5 | 7 | 7 | 8 | 6 | 6 | 7 | 7 | 6 | 8 | 10 | 8 |
| CK054_RAT | 35 | 6 | 7 | 6 | 5 | 8 | 9 | 9 | 4 | 8 | 8 | 6 | 6 |
| SIAE_RAT | 60 | 8 | 6 | 8 | 9 | 6 | 6 | 9 | 6 | 5 | 6 | 7 | 5 |
| ARK73_RAT | 37 | 7 | 9 | 0 | 3 | 10 | 11 | 4 | 7 | 5 | 6 | 5 | 6 |
| CATH_RAT | 37 | 6 | 6 | 6 | 8 | 6 | 5 | 5 | 10 | 10 | 6 | 8 | 7 |
| CD14_RAT | 40 | 5 | 7 | 8 | 7 | 7 | 8 | 9 | 6 | 8 | 9 | 7 | 9 |
| PLBL2_RAT | 65 | 6 | 4 | 7 | 4 | 6 | 5 | 5 | 6 | 4 | 7 | 7 | 6 |
| ANTR1_RAT | 62 | 5 | 4 | 4 | 7 | 7 | 7 | 7 | 4 | 5 | 4 | 4 | 7 |
| TBB5_RAT [2] | 50 | 5 | 6 | 2 | 2 | 10 | 12 | 2 | 5 | 8 | 7 | 13 | 9 |
| CO9_RAT | 62 | 6 | 6 | 7 | 8 | 4 | 7 | 5 | 9 | 8 | 4 | 8 | 9 |
| ANXA1_RAT | 39 | 9 | 7 | 4 | 7 | 5 | 5 | 4 | 2 | 9 | 8 | 7 | 10 |
| QOR_RAT | 35 | 8 | 9 | 4 | 3 | 7 | 6 | 9 | 9 | 6 | 7 | 2 | 1 |
| SO1A1_RAT | 74 | 6 | 8 | 2 | 1 | 10 | 9 | 4 | 4 | 7 | 8 | 1 | 5 |
| LHPP_RAT | 29 | 6 | 7 | 8 | 5 | 7 | 7 | 5 | 6 | 10 | 4 | 6 | 4 |
| TBA1B_RAT [2] | 50 | 3 | 5 | 2 | 4 | 7 | 9 | 0 | 2 | 4 | 9 | 14 | 7 |
| IGG2A_RAT | 35 | 7 | 3 | 11 | 9 | 8 | 8 | 11 | 13 | 8 | 9 | 8 | 14 |
| PRDX6_RAT | 25 | 3 | 6 | 4 | 5 | 4 | 8 | 5 | 6 | 8 | 4 | 4 | 6 |
| RNS1G_RAT | 17 | 6 | 6 | 3 | 1 | 8 | 6 | 9 | 10 | 8 | 5 | 8 | 8 |
| CADH2_RAT | 100 | 4 | 4 | 7 | 8 | 5 | 4 | 7 | 7 | 8 | 4 | 6 | 7 |
| OBP_RAT | 20 | 8 | 6 | 3 | 3 | 4 | 5 | 5 | 5 | 3 | 4 | 2 | 3 |
| PPBT_RAT | 58 | 6 | 8 | 2 | 4 | 5 | 6 | 2 | 7 | 7 | 6 | 7 | 6 |
| F151A_RAT | 67 | 6 | 5 | 4 | 3 | 6 | 5 | 4 | 6 | 7 | 5 | 5 | 5 |
| GRP78_RAT | 72 | 6 | 8 | 8 | 5 | 6 | 8 | 5 | 2 | 9 | 7 | 9 | 5 |
| PIP_RAT | 16 | 5 | 6 | 6 | 4 | 4 | 4 | 6 | 3 | 4 | 5 | 4 | 4 |
| THIO_RAT | 12 | 6 | 5 | 8 | 5 | 6 | 6 | 4 | 4 | 7 | 4 | 5 | 6 |
| GP2_RAT | 59 | 4 | 4 | 11 | 10 | 6 | 3 | 9 | 4 | 2 | 1 | 9 | 8 |
| GSHB_RAT | 52 | 10 | 5 | 1 | 3 | 10 | 9 | 4 | 5 | 5 | 5 | 3 | 2 |
| EHD1_RAT | 61 | 7 | 8 | 3 | 1 | 8 | 5 | 2 | 5 | 5 | 6 | 1 | 3 |
| PRDX1_RAT | 22 | 5 | 6 | 1 | 1 | 7 | 8 | 6 | 3 | 6 | 3 | 3 | 3 |
| BCAM_RAT | 68 | 6 | 6 | 4 | 8 | 2 | 6 | 6 | 5 | 6 | 5 | 3 | 7 |
| FUCO_RAT | 53 | 6 | 6 | 7 | 4 | 3 | 5 | 2 | 4 | 5 | 6 | 6 | 4 |
| TCO2_RAT | 47 | 3 | 3 | 3 | 5 | 2 | 2 | 7 | 13 | 5 | 5 | 3 | 4 |
| ATOX1_RAT | 7 | 4 | 5 | 8 | 10 | 2 | 5 | 2 | 8 | 6 | 8 | 5 | 5 |
| ACE_RAT | 151 | 6 | 3 | 3 | 1 | 6 | 5 | 2 | 2 | 4 | 5 | 6 | 3 |
| FSTL1_RAT | 35 | 4 | 3 | 7 | 4 | 4 | 3 | 7 | 4 | 5 | 4 | 6 | 6 |
| HEXA_RAT | 61 | 4 | 5 | 2 | 4 | 5 | 5 | 5 | 4 | 5 | 5 | 6 | 5 |
| NDKB_RAT [2] | 17 | 6 | 3 | 4 | 3 | 5 | 6 | 2 | 3 | 4 | 7 | 4 | 6 |
| IL1R2_RAT | 46 | 4 | 5 | 4 | 7 | 5 | 5 | 6 | 4 | 4 | 5 | 7 | 3 |
| ROB1_RAT | 26 | 4 | 6 | 4 | 4 | 5 | 6 | 6 | 6 | 5 | 5 | 6 | 5 |
| GPX3_RAT | 25 | 3 | 3 | 4 | 4 | 3 | 2 | 1 | 4 | 4 | 4 | 4 | 5 |
| NID2_RAT | 153 | 6 | 4 | 6 | 7 | 3 | 2 | 7 | 6 | 4 | 5 | 6 | 7 |
| GLNA_RAT | 42 | 4 | 5 | 1 | 0 | 7 | 8 | 1 | 4 | 3 | 5 | 3 | 3 |
| MSMB_RAT | 13 | 10 | 9 | 2 | 2 | 3 | 2 | 11 | 9 | 4 | 1 | 2 | 0 |
| S15A2_RAT | 81 | 6 | 5 | 1 | 0 | 7 | 6 | 1 | 1 | 5 | 4 | 3 | 1 |
| IDHC_RAT | 47 | 6 | 4 | 3 | 2 | 5 | 5 | 2 | 2 | 6 | 4 | 9 | 3 |
| CD166_RAT | 65 | 1 | 4 | 6 | 5 | 4 | 0 | 4 | 5 | 6 | 5 | 4 | 5 |
| GAS6_RAT | 75 | 3 | 6 | 3 | 5 | 5 | 4 | 0 | 0 | 6 | 2 | 5 | 7 |
| S7A13_RAT | 54 | 5 | 6 | 0 | 1 | 7 | 5 | 2 | 3 | 5 | 5 | 5 | 4 |
| GLCM1_RAT | 15 | 4 | 4 | 6 | 5 | 5 | 4 | 5 | 6 | 4 | 4 | 5 | 5 |
| AQP1_RAT | 29 | 5 | 6 | 3 | 2 | 6 | 4 | 4 | 3 | 2 | 2 | 3 | 3 |
| A4_RAT | 87 | 4 | 5 | 4 | 4 | 2 | 4 | 2 | 3 | 7 | 5 | 6 | 5 |
| APOA1_RAT | 30 | 5 | 6 | 7 | 7 | 4 | 3 | 6 | 2 | 2 | 1 | 4 | 2 |
| FAAA_RAT | 46 | 5 | 5 | 3 | 4 | 4 | 5 | 4 | 3 | 6 | 3 | 2 | 3 |
| IC1_RAT | 56 | 6 | 4 | 3 | 7 | 4 | 4 | 4 | 4 | 4 | 3 | 5 | 7 |
| PPT2_RAT | 34 | 6 | 3 | 6 | 4 | 5 | 3 | 6 | 4 | 4 | 3 | 5 | 3 |
| ABCG2_RAT | 73 | 7 | 6 | 0 | 0 | 7 | 7 | 1 | 2 | 5 | 6 | 3 | 4 |
| HA12_RAT | 42 | 5 | 6 | 8 | 5 | 8 | 7 | 9 | 8 | 6 | 7 | 8 | 5 |
| PGAM1_RAT | 29 | 5 | 6 | 2 | 4 | 6 | 10 | 1 | 2 | 4 | 2 | 5 | 4 |
| G6PI_RAT | 63 | 4 | 6 | 3 | 1 | 5 | 3 | 4 | 3 | 2 | 3 | 2 | 4 |
| LRC15_RAT | 64 | 1 | 3 | 19 | 12 | 2 | 1 | 1 | 1 | 0 | 0 | 1 | 1 |
| NEUR1_RAT | 45 | 6 | 5 | 4 | 3 | 1 | 2 | 7 | 2 | 4 | 3 | 2 | 2 |
| PARK7_RAT | 20 | 4 | 4 | 1 | 3 | 2 | 4 | 2 | 1 | 3 | 4 | 1 | 7 |
| LYSC2_RAT | 17 | 4 | 4 | 4 | 2 | 3 | 3 | 1 | 3 | 3 | 2 | 6 | 3 |
| GSTA1_RAT [2] | 26 | 7 | 4 | 3 | 3 | 5 | 5 | 4 | 4 | 2 | 3 | 3 | 3 |
| DOPD_RAT | 13 | 3 | 2 | 2 | 3 | 7 | 6 | 2 | 3 | 5 | 5 | 3 | 4 |
| GSTP1_RAT | 23 | 5 | 6 | 2 | 4 | 2 | 2 | 7 | 5 | 5 | 6 | 4 | 1 |
| S23A1_RAT | 65 | 3 | 5 | 0 | 1 | 5 | 6 | 1 | 0 | 3 | 6 | 7 | 6 |
| TMM27_RAT | 25 | 5 | 4 | 3 | 2 | 6 | 4 | 4 | 3 | 3 | 7 | 2 | 3 |
| ACY3_RAT | 35 | 6 | 6 | 1 | 1 | 6 | 4 | 2 | 3 | 4 | 4 | 2 | 3 |
| UK114_RAT | 14 | 5 | 4 | 3 | 3 | 4 | 3 | 4 | 5 | 2 | 3 | 2 | 3 |
| CD44_RAT | 56 | 2 | 3 | 3 | 3 | 4 | 5 | 4 | 3 | 5 | 4 | 4 | 4 |
| ALDOA_RAT | 39 | 4 | 3 | 3 | 1 | 6 | 6 | 2 | 2 | 2 | 2 | 3 | 3 |
| ASAH1_RAT | 44 | 4 | 6 | 3 | 4 | 3 | 4 | 2 | 5 | 3 | 4 | 3 | 2 |
| CD59_RAT | 14 | 4 | 3 | 6 | 3 | 3 | 3 | 6 | 4 | 3 | 5 | 3 | 4 |
| COL12_RAT | 82 | 3 | 2 | 6 | 3 | 1 | 3 | 2 | 3 | 3 | 3 | 2 | 3 |
| IL1AP_RAT | 66 | 5 | 2 | 3 | 3 | 3 | 2 | 6 | 4 | 3 | 2 | 2 | 2 |
| NAGAB_RAT | 47 | 2 | 5 | 3 | 3 | 4 | 3 | 2 | 4 | 3 | 5 | 2 | 6 |
| NEO1_RAT | 151 | 1 | 4 | 4 | 1 | 2 | 4 | 4 | 4 | 0 | 5 | 4 | 4 |
| ACTN4_RAT | 105 | 7 | 7 | 1 | 2 | 4 | 2 | 5 | 2 | 1 | 2 | 4 | 2 |
| PRSS8_RAT | 37 | 4 | 4 | 3 | 3 | 5 | 4 | 5 | 4 | 3 | 5 | 4 | 3 |
| GDIR1_RAT | 23 | 4 | 5 | 3 | 1 | 5 | 4 | 4 | 2 | 3 | 4 | 2 | 3 |
| NCAM1_RAT | 95 | 3 | 2 | 4 | 2 | 1 | 3 | 1 | 4 | 4 | 2 | 5 | 1 |
| PEPD_RAT | 55 | 4 | 4 | 0 | 2 | 4 | 3 | 1 | 2 | 2 | 4 | 6 | 2 |
| AOXD_RAT | 148 | 9 | 6 | 2 | 1 | 0 | 2 | 0 | 1 | 3 | 4 | 0 | 0 |
| PDIA1_RAT | 57 | 5 | 4 | 2 | 2 | 2 | 2 | 5 | 4 | 2 | 2 | 3 | 2 |
| PODXL_RAT | 52 | 4 | 1 | 6 | 2 | 2 | 2 | 1 | 3 | 1 | 2 | 2 | 2 |
| CTL4_RAT | 79 | 3 | 2 | 1 | 0 | 3 | 5 | 1 | 3 | 1 | 3 | 2 | 2 |
| HRG_RAT | 59 | 2 | 0 | 6 | 3 | 4 | 2 | 5 | 6 | 1 | 4 | 2 | 6 |
| ILEUA_RAT | 43 | 5 | 7 | 7 | 3 | 1 | 2 | 1 | 0 | 2 | 0 | 3 | 4 |
| KHK_RAT | 33 | 4 | 5 | 1 | 0 | 6 | 6 | 1 | 4 | 2 | 4 | 1 | 1 |
| SDCB1_RAT | 32 | 3 | 3 | 2 | 3 | 2 | 3 | 1 | 3 | 3 | 5 | 3 | 3 |
| CRYL1_RAT | 35 | 6 | 3 | 1 | 2 | 4 | 4 | 0 | 2 | 5 | 4 | 2 | 2 |
| CRYM_RAT | 34 | 4 | 4 | 2 | 1 | 6 | 3 | 0 | 1 | 5 | 7 | 2 | 2 |
| SBP1_RAT | 53 | 5 | 2 | 1 | 1 | 6 | 4 | 0 | 2 | 3 | 5 | 4 | 3 |
| SORT_RAT | 91 | 3 | 5 | 3 | 2 | 2 | 3 | 2 | 3 | 3 | 5 | 3 | 4 |
| COMP_RAT | 83 | 4 | 4 | 6 | 3 | 1 | 4 | 1 | 3 | 3 | 4 | 3 | 2 |
| LALBA_RAT | 18 | 3 | 4 | 3 | 1 | 1 | 1 | 2 | 3 | 3 | 3 | 4 | 4 |
| LDHA_RAT | 36 | 5 | 4 | 3 | 2 | 5 | 5 | 4 | 3 | 3 | 3 | 5 | 3 |
| GSTO1_RAT | 28 | 4 | 3 | 4 | 2 | 4 | 2 | 2 | 2 | 4 | 2 | 2 | 2 |
| PTER_RAT | 39 | 5 | 3 | 0 | 1 | 3 | 2 | 2 | 2 | 1 | 3 | 3 | 3 |
| RET4_RAT | 23 | 2 | 0 | 2 | 3 | 3 | 3 | 1 | 4 | 5 | 3 | 4 | 4 |
| TERA_RAT | 89 | 6 | 2 | 0 | 1 | 4 | 5 | 2 | 1 | 3 | 4 | 3 | 4 |
| SFRP4_RAT | 40 | 1 | 4 | 3 | 3 | 3 | 2 | 4 | 2 | 5 | 2 | 4 | 5 |
| ANXA4_RAT | 36 | 5 | 6 | 2 | 2 | 5 | 3 | 4 | 1 | 3 | 2 | 1 | 3 |
| LDHC_RAT | 36 | 1 | 0 | 0 | 0 | 6 | 5 | 0 | 0 | 5 | 6 | 8 | 9 |
| LITH_RAT | 19 | 3 | 1 | 4 | 5 | 2 | 1 | 1 | 3 | 3 | 1 | 6 | 5 |
| NUCB2_RAT | 50 | 2 | 3 | 2 | 2 | 2 | 2 | 0 | 2 | 5 | 5 | 5 | 2 |
| SC5A1_RAT | 73 | 3 | 5 | 0 | 0 | 4 | 5 | 0 | 0 | 5 | 3 | 2 | 1 |
| AKAP4_RAT | 93 | 0 | 0 | 0 | 0 | 12 | 17 | 0 | 0 | 0 | 0 | 0 | 0 |
| ANXA5_RAT | 36 | 3 | 2 | 1 | 1 | 4 | 2 | 4 | 4 | 1 | 5 | 3 | 5 |
| ENPP3_RAT | 99 | 5 | 2 | 2 | 3 | 3 | 4 | 2 | 1 | 2 | 1 | 4 | 1 |
| ISK1L_RAT | 9 | 1 | 5 | 3 | 4 | 6 | 5 | 6 | 8 | 0 | 0 | 0 | 0 |
| L1CAM_RAT | 141 | 1 | 3 | 2 | 5 | 1 | 3 | 2 | 3 | 1 | 3 | 1 | 3 |
| PPIB_RAT | 24 | 3 | 2 | 1 | 0 | 2 | 5 | 1 | 5 | 1 | 3 | 3 | 7 |
| PRDX5_RAT | 22 | 3 | 4 | 0 | 3 | 4 | 3 | 2 | 1 | 3 | 1 | 2 | 0 |
| S6A18_RAT | 70 | 4 | 4 | 0 | 1 | 6 | 4 | 2 | 1 | 4 | 5 | 1 | 2 |
| VAT1_RAT | 43 | 3 | 1 | 1 | 1 | 5 | 8 | 2 | 1 | 3 | 2 | 3 | 1 |
| INHBC_RAT | 39 | 3 | 4 | 3 | 2 | 3 | 3 | 2 | 4 | 2 | 3 | 3 | 4 |
| CD9_RAT | 25 | 3 | 3 | 2 | 3 | 4 | 4 | 1 | 1 | 4 | 2 | 3 | 1 |
| DHPR_RAT | 26 | 4 | 4 | 1 | 1 | 5 | 4 | 1 | 4 | 4 | 2 | 1 | 2 |
| FABPH_RAT | 15 | 2 | 2 | 3 | 2 | 3 | 4 | 5 | 2 | 3 | 1 | 6 | 3 |
| EFNA5_RAT | 26 | 1 | 2 | 2 | 2 | 3 | 3 | 5 | 3 | 3 | 2 | 2 | 2 |
| ALS_RAT | 67 | 1 | 2 | 2 | 1 | 6 | 3 | 6 | 2 | 3 | 0 | 4 | 1 |
| ATPB_RAT | 56 | 1 | 2 | 3 | 3 | 6 | 6 | 2 | 0 | 2 | 0 | 4 | 0 |
| VOME_RAT | 11 | 6 | 6 | 2 | 2 | 1 | 1 | 1 | 0 | 2 | 1 | 1 | 0 |
| ICAM1_RAT | 60 | 3 | 2 | 2 | 4 | 2 | 2 | 5 | 3 | 1 | 2 | 2 | 2 |
| SAHH_RAT | 48 | 5 | 3 | 0 | 0 | 2 | 3 | 0 | 3 | 1 | 2 | 0 | 2 |
| THY1_RAT | 18 | 3 | 2 | 3 | 2 | 2 | 1 | 1 | 3 | 3 | 2 | 2 | 1 |
| TKT_RAT | 68 | 4 | 5 | 3 | 1 | 5 | 3 | 1 | 0 | 4 | 0 | 3 | 1 |
| DDAH1_RAT | 31 | 4 | 4 | 2 | 2 | 1 | 2 | 2 | 3 | 3 | 2 | 3 | 1 |
| IGG2C_RAT | 37 | 3 | 2 | 3 | 3 | 2 | 1 | 2 | 1 | 3 | 3 | 3 | 4 |
| IST1_RAT | 40 | 3 | 3 | 2 | 2 | 2 | 2 | 4 | 2 | 2 | 2 | 2 | 3 |
| COF1_RAT | 19 | 1 | 5 | 1 | 1 | 4 | 5 | 1 | 3 | 2 | 2 | 1 | 2 |
| SDC4_RAT | 22 | 0 | 2 | 1 | 3 | 2 | 2 | 2 | 3 | 3 | 2 | 3 | 2 |
| CNTFR_RAT | 41 | 2 | 1 | 1 | 2 | 3 | 2 | 5 | 2 | 3 | 1 | 3 | 3 |
| GALM_RAT | 38 | 1 | 3 | 3 | 1 | 4 | 1 | 4 | 2 | 3 | 2 | 4 | 2 |
| CALM_RAT | 17 | 3 | 2 | 1 | 2 | 2 | 2 | 4 | 1 | 4 | 2 | 1 | 2 |
| COPB2_RAT | 103 | 3 | 1 | 3 | 2 | 1 | 0 | 2 | 2 | 1 | 3 | 2 | 3 |
| APOM_RAT | 22 | 0 | 2 | 1 | 0 | 1 | 1 | 1 | 0 | 2 | 3 | 0 | 2 |
| LGMN_RAT | 49 | 2 | 0 | 3 | 2 | 1 | 1 | 4 | 2 | 2 | 2 | 1 | 2 |
| ODFP2_RAT | 95 | 0 | 0 | 0 | 0 | 9 | 12 | 0 | 0 | 0 | 0 | 0 | 0 |
| SPA3M_RAT | 46 | 7 | 3 | 6 | 9 | 3 | 2 | 6 | 6 | 2 | 3 | 3 | 5 |
| GNAS1_RAT [4] | 123 | 4 | 4 | 0 | 0 | 8 | 5 | 0 | 1 | 1 | 1 | 1 | 1 |
| AL9A1_RAT | 54 | 2 | 6 | 2 | 2 | 4 | 3 | 4 | 0 | 2 | 0 | 2 | 0 |
| LIFR_RAT | 122 | 2 | 2 | 2 | 2 | 2 | 2 | 1 | 3 | 1 | 1 | 4 | 2 |
| MMP8_RAT | 53 | 1 | 1 | 3 | 2 | 1 | 0 | 1 | 2 | 2 | 0 | 3 | 4 |
| NIT2_RAT | 31 | 1 | 3 | 0 | 2 | 3 | 3 | 1 | 1 | 2 | 3 | 2 | 1 |
| PGFRA_RAT | 123 | 2 | 3 | 2 | 2 | 2 | 2 | 1 | 2 | 2 | 1 | 1 | 3 |
| HYAL1_RAT | 51 | 2 | 0 | 2 | 1 | 1 | 1 | 1 | 4 | 1 | 1 | 1 | 1 |
| SVS4_RAT | 12 | 0 | 0 | 3 | 2 | 3 | 3 | 5 | 3 | 2 | 1 | 3 | 3 |
| AATC_RAT | 46 | 0 | 1 | 0 | 0 | 2 | 1 | 0 | 1 | 2 | 5 | 4 | 2 |
| AT1B1_RAT | 35 | 1 | 2 | 1 | 2 | 1 | 2 | 1 | 2 | 2 | 2 | 4 | 4 |
| EF2_RAT | 95 | 2 | 2 | 0 | 0 | 4 | 6 | 1 | 1 | 1 | 2 | 1 | 1 |
| MTND_RAT | 21 | 1 | 3 | 0 | 1 | 1 | 3 | 0 | 2 | 3 | 2 | 3 | 4 |
| PLD3_RAT | 54 | 0 | 1 | 1 | 4 | 3 | 3 | 1 | 2 | 0 | 2 | 0 | 2 |
| PROF1_RAT | 15 | 2 | 3 | 1 | 1 | 3 | 4 | 0 | 3 | 1 | 3 | 1 | 2 |
| H4_RAT | 11 | 3 | 3 | 2 | 3 | 2 | 2 | 1 | 2 | 2 | 3 | 2 | 1 |
| ANXA6_RAT | 76 | 2 | 4 | 1 | 0 | 3 | 3 | 0 | 1 | 0 | 2 | 1 | 6 |
| ASGL1_RAT | 34 | 3 | 4 | 0 | 0 | 4 | 2 | 0 | 0 | 4 | 2 | 0 | 1 |
| COTL1_RAT | 16 | 3 | 2 | 1 | 1 | 1 | 2 | 1 | 1 | 1 | 2 | 1 | 1 |
| ENPP5_RAT | 54 | 4 | 4 | 1 | 0 | 1 | 0 | 2 | 3 | 1 | 0 | 1 | 1 |
| GSTM2_RAT | 26 | 1 | 2 | 2 | 0 | 5 | 3 | 1 | 1 | 4 | 0 | 2 | 2 |
| SPP24_RAT | 23 | 2 | 1 | 2 | 3 | 1 | 1 | 4 | 2 | 1 | 3 | 3 | 2 |
| TCTP_RAT | 19 | 2 | 3 | 1 | 2 | 2 | 1 | 1 | 2 | 2 | 3 | 1 | 3 |
| TYRO3_RAT | 96 | 2 | 1 | 0 | 2 | 1 | 1 | 1 | 2 | 2 | 1 | 2 | 1 |
| CYC_RAT | 12 | 3 | 3 | 1 | 1 | 2 | 1 | 1 | 1 | 2 | 2 | 2 | 4 |
| PDXK_RAT | 35 | 2 | 4 | 3 | 0 | 3 | 1 | 1 | 0 | 2 | 2 | 3 | 0 |
| CD320_RAT | 28 | 2 | 2 | 1 | 2 | 2 | 2 | 0 | 1 | 2 | 3 | 2 | 2 |
| PRIO_RAT | 28 | 2 | 3 | 1 | 1 | 2 | 2 | 2 | 1 | 3 | 1 | 3 | 2 |
| BIEA_RAT | 34 | 4 | 4 | 0 | 0 | 4 | 4 | 1 | 0 | 1 | 0 | 2 | 0 |
| CBR1_RAT | 31 | 0 | 0 | 0 | 1 | 4 | 3 | 0 | 0 | 1 | 2 | 4 | 7 |
| ENTP5_RAT | 47 | 1 | 2 | 1 | 1 | 1 | 3 | 1 | 3 | 1 | 1 | 2 | 3 |
| PEPC_RAT | 43 | 1 | 2 | 2 | 3 | 1 | 2 | 0 | 0 | 3 | 2 | 3 | 2 |
| TFF1_RAT | 9 | 3 | 1 | 3 | 1 | 2 | 2 | 1 | 1 | 3 | 2 | 1 | 2 |
| TIMP1_RAT | 24 | 3 | 2 | 1 | 0 | 5 | 4 | 1 | 0 | 1 | 2 | 1 | 1 |
| PTTG_RAT | 20 | 2 | 1 | 2 | 2 | 1 | 1 | 2 | 1 | 2 | 2 | 2 | 1 |
| FURIN_RAT | 87 | 4 | 1 | 1 | 0 | 1 | 0 | 2 | 1 | 1 | 2 | 1 | 0 |
| GPC5C_RAT | 48 | 1 | 2 | 1 | 3 | 2 | 2 | 0 | 2 | 1 | 3 | 1 | 3 |
| MYO1D_RAT | 116 | 2 | 1 | 0 | 0 | 6 | 3 | 0 | 0 | 0 | 0 | 1 | 0 |
| IAH1_RAT | 28 | 3 | 2 | 1 | 1 | 1 | 2 | 1 | 2 | 2 | 2 | 1 | 2 |
| ANXA2_RAT | 39 | 3 | 1 | 1 | 0 | 3 | 0 | 2 | 1 | 2 | 0 | 2 | 1 |
| CSAD_RAT | 55 | 3 | 3 | 0 | 0 | 4 | 2 | 2 | 2 | 1 | 0 | 0 | 1 |
| GALNS_RAT | 58 | 2 | 2 | 1 | 1 | 1 | 3 | 2 | 3 | 0 | 0 | 2 | 0 |
| PPT1_RAT | 34 | 3 | 1 | 1 | 2 | 1 | 0 | 1 | 5 | 2 | 3 | 1 | 1 |
| R4RL2_RAT | 46 | 1 | 1 | 2 | 2 | 1 | 1 | 4 | 3 | 1 | 0 | 2 | 2 |
| TIMD2_RAT | 39 | 2 | 1 | 2 | 1 | 2 | 2 | 2 | 0 | 2 | 2 | 1 | 2 |
| DEST_RAT | 19 | 3 | 1 | 0 | 0 | 4 | 4 | 2 | 1 | 2 | 1 | 1 | 0 |
| DHSO_RAT | 38 | 0 | 3 | 1 | 0 | 1 | 4 | 0 | 1 | 0 | 0 | 1 | 0 |
| GPM6A_RAT | 31 | 2 | 2 | 1 | 0 | 3 | 3 | 1 | 1 | 2 | 2 | 1 | 1 |
| KAT1_RAT | 52 | 2 | 1 | 1 | 0 | 3 | 2 | 0 | 0 | 3 | 1 | 1 | 1 |
| PTGR1_RAT | 36 | 1 | 3 | 0 | 1 | 0 | 0 | 1 | 1 | 1 | 1 | 0 | 1 |
| PAG15_RAT | 47 | 2 | 1 | 1 | 2 | 1 | 1 | 1 | 2 | 0 | 0 | 1 | 1 |
| ENPP6_RAT | 51 | 3 | 2 | 1 | 0 | 2 | 1 | 2 | 1 | 1 | 0 | 1 | 1 |
| GBB1_RAT | 37 | 6 | 6 | 1 | 2 | 6 | 6 | 2 | 2 | 4 | 4 | 4 | 1 |
| GPX41_RAT (+1) | 22 | 1 | 0 | 0 | 0 | 5 | 5 | 0 | 1 | 0 | 1 | 0 | 0 |
| MFGM_RAT | 47 | 1 | 2 | 0 | 0 | 0 | 2 | 0 | 1 | 5 | 2 | 2 | 0 |
| ODFP1_RAT | 27 | 0 | 0 | 0 | 0 | 8 | 6 | 0 | 0 | 0 | 0 | 0 | 0 |
| RHOA_RAT | 22 | 1 | 1 | 0 | 0 | 3 | 2 | 0 | 0 | 0 | 2 | 0 | 2 |
| PLOD3_RAT | 85 | 1 | 2 | 2 | 1 | 2 | 1 | 0 | 1 | 1 | 2 | 2 | 3 |
| MIA_RAT | 15 | 1 | 2 | 0 | 2 | 2 | 1 | 0 | 1 | 1 | 1 | 0 | 2 |
| BASP1_RAT | 22 | 0 | 0 | 0 | 0 | 3 | 3 | 1 | 2 | 2 | 2 | 1 | 1 |
| LAMP1_RAT | 44 | 2 | 3 | 1 | 2 | 0 | 0 | 2 | 3 | 1 | 1 | 1 | 0 |
| RB11A_RAT (+1) | 24 | 1 | 2 | 1 | 0 | 4 | 2 | 1 | 1 | 2 | 0 | 1 | 0 |
| THNS2_RAT | 54 | 3 | 4 | 1 | 0 | 1 | 2 | 0 | 0 | 1 | 2 | 1 | 0 |
| CADM3_RAT | 43 | 2 | 1 | 1 | 1 | 1 | 1 | 1 | 3 | 0 | 2 | 2 | 2 |
| SHPS1_RAT | 56 | 1 | 0 | 2 | 2 | 2 | 1 | 2 | 2 | 2 | 1 | 1 | 0 |
| HAOX2_RAT | 39 | 3 | 3 | 0 | 0 | 2 | 3 | 0 | 2 | 1 | 2 | 0 | 1 |
| IGG2B_RAT | 36 | 1 | 1 | 1 | 1 | 3 | 1 | 1 | 2 | 1 | 1 | 2 | 2 |
| KNG1_RAT | 71 | 1 | 2 | 3 | 2 | 3 | 2 | 2 | 2 | 3 | 1 | 4 | 2 |
| MDHM_RAT | 36 | 1 | 1 | 2 | 1 | 0 | 1 | 1 | 2 | 0 | 3 | 2 | 1 |
| DFB50_RAT | 8 | 2 | 3 | 0 | 0 | 1 | 2 | 2 | 4 | 0 | 0 | 1 | 0 |
| VCAM1_RAT | 81 | 1 | 1 | 1 | 2 | 2 | 1 | 4 | 0 | 1 | 0 | 2 | 0 |
| VAS1_RAT | 51 | 1 | 2 | 2 | 1 | 1 | 0 | 1 | 0 | 0 | 0 | 1 | 1 |
| CXAR_RAT | 40 | 0 | 1 | 0 | 1 | 0 | 0 | 1 | 0 | 2 | 1 | 1 | 1 |
| AL1A1_RAT | 54 | 0 | 2 | 0 | 2 | 2 | 3 | 0 | 0 | 0 | 1 | 2 | 0 |
| BHMT2_RAT | 40 | 1 | 3 | 0 | 0 | 3 | 3 | 0 | 1 | 0 | 0 | 1 | 1 |
| ESTD_RAT | 31 | 2 | 3 | 0 | 1 | 3 | 1 | 1 | 0 | 1 | 0 | 1 | 0 |
| GBB2_RAT | 37 | 5 | 4 | 1 | 2 | 7 | 4 | 2 | 4 | 3 | 7 | 4 | 1 |
| KAP2_RAT | 46 | 1 | 1 | 0 | 0 | 3 | 1 | 0 | 0 | 0 | 1 | 1 | 2 |
| AMPL_RAT | 56 | 0 | 0 | 0 | 0 | 0 | 3 | 0 | 0 | 0 | 3 | 0 | 1 |
| ITIH3_RAT | 99 | 1 | 2 | 1 | 2 | 1 | 0 | 2 | 2 | 1 | 2 | 1 | 1 |
| OX2G_RAT | 31 | 1 | 1 | 2 | 2 | 2 | 1 | 2 | 2 | 1 | 0 | 1 | 1 |
| S10AB_RAT | 11 | 1 | 1 | 2 | 1 | 1 | 2 | 2 | 1 | 1 | 1 | 1 | 1 |
| GPX1_RAT | 22 | 2 | 0 | 0 | 0 | 0 | 2 | 0 | 0 | 0 | 2 | 1 | 1 |
| H2A1C_RAT (+7) | 14 | 1 | 0 | 1 | 0 | 0 | 1 | 0 | 0 | 0 | 0 | 2 | 1 |
| CALR_RAT | 48 | 0 | 0 | 0 | 0 | 2 | 4 | 0 | 0 | 2 | 1 | 1 | 1 |
| STXB2_RAT | 67 | 1 | 2 | 0 | 0 | 2 | 4 | 0 | 0 | 0 | 2 | 0 | 0 |
| EFNA1_RAT | 24 | 0 | 0 | 2 | 0 | 1 | 0 | 2 | 1 | 0 | 0 | 0 | 0 |
| TIP_RAT | 67 | 2 | 1 | 1 | 1 | 1 | 1 | 1 | 2 | 0 | 0 | 1 | 1 |
| ACV1B_RAT | 57 | 0 | 0 | 2 | 2 | 0 | 0 | 0 | 0 | 3 | 3 | 3 | 2 |
| NTF2_RAT | 14 | 1 | 1 | 1 | 1 | 1 | 1 | 1 | 0 | 2 | 1 | 2 | 1 |
| OAF_RAT | 32 | 0 | 0 | 1 | 1 | 0 | 1 | 1 | 1 | 1 | 2 | 0 | 2 |
| 4F2_RAT | 58 | 1 | 1 | 1 | 1 | 1 | 1 | 0 | 1 | 0 | 1 | 2 | 2 |
| GDIA_RAT | 51 | 2 | 6 | 1 | 4 | 3 | 3 | 0 | 2 | 2 | 1 | 3 | 0 |
| IGHG1_RAT | 36 | 4 | 3 | 4 | 4 | 0 | 2 | 6 | 6 | 2 | 5 | 3 | 4 |
| TIMP2_RAT | 24 | 2 | 1 | 0 | 0 | 0 | 1 | 1 | 0 | 1 | 0 | 0 | 0 |
| APOC1_RAT | 10 | 2 | 1 | 1 | 3 | 1 | 1 | 1 | 0 | 1 | 0 | 1 | 0 |
| SUIS_RAT | 210 | 1 | 2 | 0 | 1 | 1 | 2 | 1 | 0 | 2 | 1 | 1 | 0 |
| RENR_RAT | 39 | 0 | 2 | 2 | 1 | 2 | 2 | 0 | 0 | 1 | 1 | 2 | 0 |
| GSTM1_RAT | 26 | 1 | 1 | 0 | 0 | 4 | 0 | 1 | 0 | 1 | 1 | 1 | 1 |
| GSTM5_RAT | 27 | 0 | 0 | 0 | 0 | 5 | 5 | 0 | 0 | 0 | 0 | 0 | 0 |
| APLP2_RAT | 87 | 1 | 0 | 0 | 1 | 0 | 0 | 0 | 1 | 1 | 1 | 1 | 3 |
| EST4_RAT | 62 | 1 | 1 | 0 | 1 | 0 | 0 | 1 | 3 | 0 | 0 | 0 | 3 |
| K1C24_RAT | 52 | 0 | 0 | 0 | 0 | 2 | 2 | 0 | 0 | 0 | 0 | 0 | 0 |
| ASM3A_RAT | 50 | 1 | 0 | 2 | 1 | 1 | 2 | 1 | 0 | 1 | 0 | 1 | 0 |
| CD82_RAT | 29 | 1 | 1 | 0 | 0 | 2 | 1 | 1 | 1 | 1 | 0 | 1 | 2 |
| MA2A1_RAT | 131 | 1 | 1 | 0 | 0 | 1 | 1 | 2 | 1 | 1 | 1 | 2 | 0 |
| MSRA_RAT | 26 | 2 | 0 | 0 | 0 | 0 | 1 | 0 | 0 | 1 | 1 | 1 | 0 |
| CTHR1_RAT | 26 | 1 | 1 | 1 | 2 | 1 | 1 | 1 | 1 | 1 | 1 | 1 | 1 |
| HYOU1_RAT | 111 | 1 | 0 | 1 | 0 | 1 | 1 | 0 | 0 | 2 | 1 | 1 | 1 |
| GRPB_RAT | 27 | 0 | 0 | 0 | 0 | 2 | 3 | 0 | 0 | 0 | 0 | 2 | 1 |
| H2B1_RAT | 14 | 2 | 1 | 3 | 2 | 3 | 2 | 1 | 2 | 2 | 2 | 1 | 2 |
| MUC18_RAT | 71 | 0 | 1 | 1 | 0 | 1 | 0 | 4 | 1 | 1 | 0 | 1 | 0 |
| TRXR1_RAT | 55 | 2 | 2 | 0 | 0 | 3 | 1 | 0 | 1 | 1 | 1 | 1 | 0 |
| FAM3C_RAT | 25 | 0 | 1 | 2 | 1 | 0 | 0 | 0 | 0 | 1 | 0 | 2 | 0 |
| AMD_RAT | 109 | 0 | 0 | 0 | 2 | 0 | 0 | 1 | 1 | 0 | 2 | 2 | 1 |
| CNDP1_RAT | 55 | 3 | 1 | 0 | 0 | 2 | 2 | 0 | 0 | 1 | 0 | 1 | 0 |
| PCOC1_RAT | 50 | 1 | 2 | 0 | 1 | 0 | 1 | 0 | 2 | 1 | 0 | 1 | 1 |
| CMBL_RAT | 28 | 1 | 1 | 1 | 0 | 0 | 2 | 0 | 0 | 1 | 1 | 1 | 1 |
| FPRP_RAT | 99 | 0 | 1 | 0 | 2 | 0 | 2 | 0 | 0 | 0 | 0 | 2 | 1 |
| RAP1A_RAT | 21 | 1 | 0 | 1 | 1 | 2 | 1 | 1 | 0 | 1 | 1 | 1 | 1 |
| RAB14_RAT | 24 | 2 | 1 | 1 | 0 | 2 | 1 | 2 | 0 | 1 | 0 | 0 | 0 |
| ASPG_RAT | 37 | 2 | 2 | 1 | 1 | 1 | 1 | 2 | 0 | 0 | 0 | 0 | 0 |
| ANGL4_RAT | 45 | 2 | 2 | 1 | 1 | 0 | 0 | 0 | 1 | 1 | 2 | 0 | 0 |
| DLDH_RAT | 54 | 1 | 1 | 0 | 0 | 0 | 1 | 0 | 0 | 0 | 1 | 2 | 2 |
| CFAI_RAT | 67 | 0 | 0 | 3 | 0 | 1 | 0 | 1 | 0 | 2 | 0 | 0 | 0 |
| CBPB2_RAT | 49 | 0 | 0 | 0 | 1 | 2 | 1 | 1 | 0 | 0 | 1 | 0 | 1 |
| PRDX2_RAT | 22 | 2 | 1 | 0 | 1 | 0 | 0 | 1 | 0 | 1 | 2 | 0 | 0 |
| AL3A1_RAT | 50 | 5 | 4 | 0 | 0 | 0 | 0 | 0 | 0 | 0 | 0 | 0 | 0 |
| BAIP2_RAT | 59 | 0 | 0 | 0 | 0 | 2 | 3 | 0 | 0 | 1 | 0 | 0 | 0 |
| HBA_RAT | 15 | 0 | 0 | 0 | 0 | 1 | 4 | 0 | 0 | 0 | 0 | 0 | 0 |
| STC1_RAT | 28 | 0 | 1 | 0 | 1 | 1 | 0 | 1 | 3 | 2 | 0 | 1 | 0 |
| AGRL1_RAT | 167 | 3 | 0 | 1 | 0 | 2 | 0 | 1 | 0 | 0 | 1 | 0 | 0 |
| ITM2B_RAT | 30 | 2 | 0 | 0 | 0 | 0 | 2 | 0 | 0 | 2 | 1 | 1 | 0 |
| CADM4_RAT | 43 | 0 | 1 | 2 | 1 | 0 | 1 | 0 | 2 | 0 | 1 | 0 | 0 |
| CATZ_RAT | 34 | 0 | 1 | 0 | 0 | 1 | 1 | 1 | 1 | 0 | 1 | 2 | 1 |
| CD302_RAT | 25 | 1 | 2 | 1 | 1 | 0 | 1 | 1 | 0 | 1 | 0 | 2 | 0 |
| GDF15_RAT | 33 | 1 | 1 | 0 | 0 | 1 | 0 | 0 | 1 | 2 | 2 | 1 | 1 |
| S22AC_RAT | 60 | 1 | 1 | 0 | 0 | 2 | 2 | 0 | 0 | 2 | 0 | 0 | 1 |
| GLO2_RAT | 34 | 0 | 2 | 0 | 0 | 1 | 2 | 2 | 0 | 1 | 1 | 1 | 0 |
| PNPH_RAT | 32 | 1 | 1 | 0 | 1 | 0 | 1 | 0 | 0 | 1 | 2 | 1 | 1 |
| PRDX3_RAT | 28 | 1 | 2 | 1 | 1 | 0 | 0 | 0 | 1 | 0 | 1 | 0 | 2 |
| GPC1_RAT | 62 | 0 | 0 | 0 | 1 | 1 | 0 | 0 | 0 | 0 | 1 | 1 | 2 |
| F213A_RAT | 26 | 0 | 0 | 0 | 0 | 1 | 0 | 0 | 0 | 2 | 0 | 4 | 1 |
| FABP9_RAT | 15 | 0 | 0 | 0 | 0 | 5 | 3 | 0 | 0 | 1 | 0 | 0 | 0 |
| G3PT_RAT | 47 | 3 | 2 | 2 | 0 | 6 | 3 | 1 | 2 | 3 | 1 | 2 | 2 |
| IBP3_RAT | 32 | 0 | 1 | 0 | 0 | 1 | 0 | 0 | 0 | 2 | 1 | 1 | 1 |
| ATPA_RAT | 60 | 0 | 0 | 0 | 0 | 1 | 4 | 0 | 0 | 0 | 0 | 0 | 0 |
| CYC2_RAT | 12 | 0 | 0 | 0 | 0 | 0 | 0 | 0 | 0 | 1 | 1 | 2 | 4 |
| CDC42_RAT | 21 | 0 | 2 | 1 | 0 | 2 | 1 | 0 | 0 | 1 | 0 | 1 | 0 |
| GDN_RAT | 44 | 0 | 0 | 0 | 0 | 0 | 1 | 2 | 2 | 0 | 1 | 0 | 0 |
| APT_RAT | 20 | 0 | 0 | 2 | 0 | 3 | 1 | 0 | 0 | 0 | 0 | 0 | 0 |
| FZD2_RAT | 64 | 0 | 0 | 1 | 0 | 2 | 0 | 0 | 0 | 2 | 3 | 3 | 1 |
| RNS10_RAT | 24 | 0 | 0 | 0 | 0 | 1 | 1 | 0 | 0 | 2 | 0 | 1 | 0 |
| FGL1_RAT | 36 | 0 | 2 | 0 | 0 | 0 | 1 | 1 | 0 | 0 | 1 | 0 | 1 |
| KCY_RAT | 22 | 0 | 2 | 0 | 2 | 2 | 0 | 1 | 0 | 1 | 0 | 0 | 0 |
| PGM1_RAT | 61 | 0 | 1 | 0 | 0 | 0 | 1 | 1 | 0 | 2 | 0 | 0 | 0 |
| GNA11_RAT | 42 | 1 | 0 | 0 | 0 | 2 | 0 | 0 | 0 | 0 | 0 | 0 | 0 |
| S22A5_RAT | 63 | 1 | 1 | 0 | 0 | 2 | 1 | 0 | 0 | 1 | 0 | 1 | 0 |
| SO4C1_RAT | 79 | 1 | 1 | 0 | 0 | 2 | 0 | 0 | 0 | 0 | 0 | 0 | 0 |
| REG3B_RAT | 20 | 0 | 0 | 2 | 2 | 0 | 0 | 0 | 0 | 1 | 0 | 2 | 0 |
| SG2A1_RAT | 11 | 2 | 1 | 0 | 0 | 1 | 0 | 0 | 0 | 1 | 0 | 1 | 0 |
| 5NTD_RAT | 64 | 0 | 2 | 0 | 0 | 0 | 0 | 0 | 2 | 0 | 0 | 0 | 1 |
| CSF1_RAT | 62 | 0 | 0 | 0 | 0 | 0 | 0 | 0 | 0 | 1 | 0 | 3 | 0 |
| SVS5_RAT | 14 | 0 | 0 | 0 | 0 | 1 | 1 | 2 | 0 | 0 | 0 | 0 | 0 |
| KIRR1_RAT | 87 | 2 | 0 | 0 | 1 | 0 | 0 | 0 | 0 | 1 | 1 | 0 | 1 |
| CO1A1_RAT | 138 | 0 | 2 | 0 | 0 | 0 | 0 | 0 | 0 | 0 | 0 | 1 | 1 |
| SC5A2_RAT | 73 | 0 | 2 | 0 | 0 | 1 | 2 | 0 | 0 | 0 | 0 | 0 | 0 |
| HS90B_RAT | 83 | 0 | 0 | 0 | 0 | 2 | 1 | 0 | 0 | 2 | 2 | 2 | 1 |
| HPPD_RAT | 45 | 1 | 2 | 0 | 0 | 0 | 0 | 0 | 0 | 0 | 0 | 0 | 0 |
| JAM3_RAT | 35 | 0 | 0 | 1 | 1 | 0 | 0 | 0 | 2 | 0 | 0 | 1 | 0 |
| THTM_RAT | 33 | 0 | 0 | 0 | 0 | 0 | 1 | 0 | 0 | 0 | 0 | 1 | 2 |
| ENPL_RAT | 93 | 0 | 0 | 0 | 0 | 2 | 2 | 0 | 0 | 3 | 1 | 1 | 0 |
| ACY2_RAT | 35 | 2 | 0 | 0 | 0 | 1 | 0 | 0 | 0 | 0 | 1 | 0 | 0 |
| RASH_RAT (+1) | 21 | 0 | 0 | 0 | 0 | 1 | 2 | 0 | 0 | 0 | 0 | 1 | 0 |
| SNAA_RAT | 33 | 0 | 1 | 0 | 0 | 2 | 1 | 0 | 0 | 0 | 0 | 0 | 0 |
| CAZA2_RAT | 33 | 0 | 2 | 0 | 0 | 0 | 0 | 0 | 0 | 1 | 0 | 0 | 1 |
| COX2_RAT | 26 | 0 | 0 | 0 | 0 | 2 | 1 | 0 | 0 | 0 | 0 | 1 | 0 |
| LIPE_RAT | 56 | 0 | 0 | 0 | 0 | 2 | 1 | 0 | 0 | 0 | 0 | 0 | 0 |
| DNJC5_RAT | 22 | 0 | 0 | 0 | 0 | 0 | 2 | 0 | 0 | 0 | 1 | 0 | 0 |
| NICA_RAT | 78 | 2 | 0 | 0 | 0 | 1 | 0 | 0 | 0 | 0 | 0 | 0 | 0 |
| FZD8_RAT | 73 | 0 | 0 | 0 | 0 | 0 | 0 | 0 | 0 | 0 | 0 | 0 | 2 |
| ACSL4_RAT | 74 | 1 | 0 | 0 | 0 | 2 | 0 | 0 | 0 | 0 | 0 | 0 | 0 |
| H2B1A_RAT | 14 | 1 | 0 | 2 | 0 | 1 | 1 | 1 | 0 | 2 | 2 | 0 | 1 |
| ECHA_RAT | 83 | 0 | 0 | 0 | 0 | 2 | 0 | 0 | 0 | 0 | 0 | 0 | 0 |
| FIBA_RAT | 87 | 0 | 0 | 2 | 0 | 0 | 0 | 0 | 0 | 0 | 0 | 0 | 0 |

Note: NS refers to the normal saline treatment group, ES refers to the etamsylate treatment group, and 1_1 and 1_2 refer to two technical repetitions of the same rat. For example: NS 1_1 refers to the first technical repetition of the first rat in the saline group.
